# HERC2 promotes inflammation-driven cancer stemness and immune evasion in hepatocellular carcinoma by activating STAT3 pathway

**DOI:** 10.1186/s13046-023-02609-0

**Published:** 2023-02-01

**Authors:** Yunzhi Liu, Qishan Xu, Fan Deng, Zhuojun Zheng, Jialiang Luo, Ping Wang, Jia Zhou, Xiao Lu, Liyun Zhang, Zhengliang Chen, Qifan Zhang, Qingyun Chen, Daming Zuo

**Affiliations:** 1 Department of Medical Laboratory, School of Laboratory Medicine and Biotechnology, Southern Medical University, Guangzhou, 510515 Guangdong China; 2 Guangdong Province Key Laboratory of Proteomics, Department of Immunology, School of Basic Medical Sciences, Southern Medical University, Guangzhou, 510515 Guangdong China; 3 Clinical Oncology Center, Shenzhen Key Laboratory for Cancer Metastasis and Personalized Therapy, The University of Hong Kong-Shenzhen Hospital, Shenzhen, 518053 Guangdong China; 4 Shenzhen Institute of Advanced Technology, Chinese Academy of Sciences, Shenzhen, 518055 China; 5 Department of Hepatobiliary Surgery, Nanfang Hospital, Southern Medical University, Guangzhou, 510515 Guangdong China; 6 Medical Research Institute, Guangdong Provincial People’s Hospital, Guangdong Academy of Medical Sciences, Guangzhou, 510080 Guangdong China; 7 Guangdong Province Key Laboratory of Immune Regulation and Immunotherapy, School of Laboratory Medicine and Biotechnology, Southern Medical University, Guangzhou, 510515 Guangdong China; 8 Microbiome Medicine Center, Zhujiang Hospital, Southern Medical University, Guangzhou, 510282 Guangdong China

**Keywords:** HERC2, Hepatocellular carcinoma, JAK2/STAT3 signaling, Cancer stemness, PD-L1

## Abstract

**Background:**

Hepatic inflammation is a common initiator of liver diseases and considered as the primary driver of hepatocellular carcinoma (HCC). However, the precise mechanism of inflammation-induced HCC development and immune evasion remains elusive and requires extensive investigation. This study sought to identify the new target that is involved in inflammation-related liver tumorigenesis.

**Methods:**

RNA-sequencing (RNA-seq) analysis was performed to identify the differential gene expression signature in primary human hepatocytes treated with or without inflammatory stimulus. A giant E3 ubiquitin protein ligase, HECT domain and RCC1-like domain 2 (HERC2), was identified in the analysis. Prognostic performance in the TCGA validation dataset was illustrated by Kaplan–Meier plot. The functional role of HERC2 in HCC progression was determined by knocking out and over-expressing HERC2 in various HCC cells. The precise molecular mechanism and signaling pathway networks associated with HERC2 in HCC stemness and immune evasion were determined by quantitative real-time PCR, immunofluorescence, western blot, and transcriptomic profiling analyses. To investigate the role of HERC2 in the etiology of HCC in vivo, we applied the chemical carcinogen diethylnitrosamine (DEN) to hepatocyte-specific HERC2-knockout mice. Additionally, the orthotopic transplantation mouse model of HCC was established to determine the effect of HERC2 during HCC development.

**Results:**

We found that increased HERC2 expression was correlated with poor prognosis in HCC patients. HERC2 enhanced the stemness and PD-L1-mediated immune evasion of HCC cells, which is associated with the activation of signal transducer and activator of transcription 3 (STAT3) pathway during the inflammation-cancer transition. Mechanically, HERC2 coupled with the endoplasmic reticulum (ER)-resident protein tyrosine phosphatase 1B (PTP1B) and limited PTP1B translocation from ER to ER-plasma membrane junction, which ameliorated the inhibitory role of PTP1B in Janus kinase 2 (JAK2) phosphorylation. Furthermore, HERC2 knockout in hepatocytes limited hepatic PD-L1 expression and ameliorated HCC progression in DEN-induced mouse liver carcinogenesis. In contrast, HERC2 overexpression promoted tumor development and progression in the orthotopic transplantation HCC model.

**Conclusion:**

Our data identified HERC2 functions as a previously unknown modulator of the JAK2/STAT3 pathway, thereby promoting inflammation-induced stemness and immune evasion in HCC.

**Supplementary Information:**

The online version contains supplementary material available at 10.1186/s13046-023-02609-0.

## Background

Nonresolving chronic inflammation is associated with persistent hepatic injury and concurrent regeneration, leading to liver fibrosis and cirrhosis thereafter contributing to the development and progression of hepatocellular carcinoma (HCC) [1–3]. The inflammatory responses play crucial roles at all stages of HCC development, including initiation, promotion, malignant conversion, invasion, and metastasis. A growing number of preclinical and clinical studies have identified a plethora of inflammatory mediators and signaling pathways implicated in HCC [1, 4, 5]. Elevated serum levels of IL-6 have been found to be associated with an increased risk of HCC development in patients with chronic hepatitis B and C infections [6]. It has also been determined that obesity-promoted HCC development is associated with enhanced production of inflammatory cytokines IL-6 and TNF-α, which is dependent on the activation of signal transducer and activator of transcription 3 (STAT3) [7]. Of note, the inflammatory cytokine microenvironment promotes retrodifferentiation and cancer stemness, which are closely associated with HCC initiation and enable the malignant features of HCC [8]. Accumulating evidence has shown that cancer stem cells (CSCs) are closely related to HCC initiation and promote the malignant features of HCC, making them a promising target for developing novel anti-cancer drugs [9]. Hence, further investigation of the correlation between cancer stemness and cytokine-mediated inflammation will broaden our understanding of the pathogenesis and therapy of HCCs.

Immune evasion from cytotoxic immune cells is essential for cancer initiation and later metastasis. However, its dynamics at intermediate stages, where potential therapeutic interventions might be carried out, are not well defined. It should be mentioned that progressive immune evasion is validated in treatment-naïve patients with stage I to III HCC and a murine model of HCC [10]. Chronic inflammation in the tumor microenvironment impairs cytotoxic T lymphocyte activation and promotes immunosuppression [11]. Programmed death ligand-1 (PD-L1) is an essential immune checkpoint protein whose overexpression on tumor cells provides a mechanism to escape immune surveillance. Lim et al*.*, observed that cancer-related inflammation is able to increase and stabilize protein expression of PD-L1 on tumor cells [12]. Interestingly, cancer stemness has also been shown to be greatly associated with tumor-intrinsic immunosuppressive features. A previous study identified regulators in liver CSCs that can modulate the expression of PD-L1, indicating a possible immune evasion mechanism initiated by CSCs [13]. PD-1/PD-L1 blockade cancer immunotherapy is increasingly used for the treatment of advanced HCC and is associated with an overall survival benefit [14, 15]. Therefore, the continued characterization of the novel connection between inflammation and tumor immune evasion and identification of crucial inflammatory events with PD-L1 expression in HCC would provide additional targets for improving the clinical responses with immune checkpoint blockade therapies.

HECT and RLD domain-containing E3 ubiquitin-protein ligase 2 (HERC2) belongs to the large HERC family of ubiquitin E3 ligases with multiple structural domains that have been implicated in a wide range of physiological processes, including membrane trafficking, immune response, DNA repair, inflammation, cell stress response, and cancer biology [16–18]. Indeed, HERC2 is related to the pathogenesis of several inflammatory and autoimmune diseases, like inflammatory bowel diseases, type 1 diabetes, and sarcoidosis [16]. However, the role and function of HERC2 in inflammation-related HCC progression are still unknown. Following our initial study showing HERC2 induction upon inflammatory stimulation in human primary hepatocytes, we explored the role of HERC2 in the progression of inflammation-associated HCC progression. The result showed that HERC2 expression in hepatocytes was associated with the progression and poor prognosis of HCC. Next, we determined that HERC2 promoted the malignant phenotype and stemness of HCC cells through the Janus kinase 2 (JAK2)/STAT3 signaling pathway. Moreover, HERC2 deficiency can decrease T cell exhaustion by modulating the STAT3-induced PD-L1 expression. Overall, the role of HERC2 in HCC provides new insight into the relationship between inflammatory processes and liver tumorigenicity and indicates that HERC2 could be a potential HCC therapeutic target.

## Methods

### Human clinical samples

Human HCC tissues and paired adjacent non-tumor tissues were acquired from patients at Nanfang Hospital, Southern Medical University. The adjacent tissues were at least 2 cm away from the paired HCC tissues. The investigation was approved by the Medical Ethics Committee of Southern Medical University (NFEC-2017–119).

For the immunohistochemistry assay, tissues were fixed in 4% paraformaldehyde for further study.

The isolated non-tumor liver tissues were cut into small pieces and digested with 1 mg/ml type IV collagenase and DNAase at 37 °C for 30 min. The cell suspension was then passed through a 70 μm filter and centrifuged at 4 °C and 50 g/min for 1 min. The cells were then washed three times with cold PBS at 4 °C and 50 g/min for 1 min. After the final centrifugation, the cells were removed upon 70% Percoll and centrifuged at 22 °C and 400 g/min for 10 min. The cells were then washed three times with cold PBS at 4 °C and collected for further analyses.

### Bioinformatics assay

For bulk RNA-seq analysis, all datasets involved in this study were available from Gene Expression Omnibus (GEO) (GSE25097, GSE14520) website and The Cancer Genome Atlas (TCGA) official website LIHC project. The samples have been divided into two groups based on the median expression among HCC and adjacent liver samples. For survival analysis, survival data of a cohort composed of patients with cirrhosis backgrounds from GSE14520 and TCGA datasets were analyzed. In GSE14520 datasets, some samples in the datasets missed the adjacent paired counterparts or AFP level, thus 198 paired samples were included for paired comparison and 200 samples were conducted for AFP level analysis.

For single cell RNA-seq analysis, samples were obtained from GSE146115 datasets. To analyze single cell data, Seurat object was created by Seurat R package (version 3.0). The cells with unique feature counts from 200–5000, or < 25% mitochondrial counts have remained. The remaining cells were clustered based on UMAP. Tumor cells were then included for further analysis. Among the tumor cell subsets, cells with HERC2 gene counts > 0 were identified as HERC2-positive cells. IL-6-JAK-STAT3 pathway-related genes were obtained from GSEA datasets (http://www.gsea-msigdb.org/gsea/index.jsp) (HALLMARK IL6 JAK STAT3 SIGNALING, CHIP-seq from STAT3_01).

### Establishment of hepatocyte-specific HERC2 knockout mice

The HERC2 targeting construct was linearized by restriction digestion with NotI followed by phenol/chloroform extraction and ethanol precipitation. The linearized vector was transfected into C57BL/6N embryonic stem (ES) cells by electroporation. The transfected ES cells were subject to G418 selection (200 μg/ml) 24 h post-electroporation. 186 G418 resistant clones were picked and amplified. Then, clones were used for homologous recombination. The PCR screening identified forty-five potential targeted clones, from among which six were expanded and further characterized by Southern blot analysis. Five of the six expanded clones were confirmed to be correctly targeted prior to blastocyst injection (as shown in supplemental Fig. 5A). The resulting pups were backcrossed to C57BL/6N or F/Cre mice at 8-week-old. The genotype was obtained from tail sniping.

### Animal breeding and treatments

Mice had free access to water and commercial feed and were kept under a 12 h light/dark cycle. Mice were housed at a constant temperature (19–23 °C) and 55 ± 10% humidity. All animal experiments were approved by the Welfare and Ethical Committee for Experimental Animal Care of Southern Medical University (2020066). All mice were euthanized with 5% isoflurane. For mouse inflammation-related HCC model induction, 15-day-old male mice were intraperitoneally injected with 25 μg/g diethylnitrosamine (DEN). Two weeks later, the mice were intraperitoneally injected with 0.5 μl/g carbon tetrachloride (CCl4) once a week for consecutive 22 weeks. For the mouse orthotopic injection HCC model, 2 × 10^6^ or 2 × 10^5^ HERC2-overexpressing Hepa1-6 cells or control cells were orthotopically injected into the livers of 6-week-old wild-type C57BL/6 J male mice for 4 weeks.

### Cell culture and treatments

The cell lines involved in this study have been performed short tandem repeat (STR) profiling to guarantee authenticity. Moreover, all the cell lines have been tested for mycoplasma negative based on PCR analysis (C0301S, Beyotime Biotechnology, Shanghai, China). Cells were cultured in DMEM (11,965,092, Thermo Fisher Scientific, Inc, CA, USA) supplemented with 10% fetal bovine serum (FBS), 100 U/ml penicillin, and 100 μg/ml streptomycin. Cells were maintained at 37 °C and 5% CO_2_. Plasmids were transfected through Lipo3000 (L3000150, Thermo Fisher Scientific, Inc.) based on the instructions.

### CCK-8 assay

Cell proliferation and viability were tested by Cell Counting Kit-8 (CCK-8, Dojindo Molecular Technologies, Japan) based on the manufacturer’s protocols. Briefly, cells were seeded into 96-well plates. 10 μl of CCK8 solution were then added to the culture medium, followed by incubation at 37 °C for 2 h. The 450 nm absorbance was detected.

### Colony formation assay

Cells were seeded into 6-well plates. After being cultured for 7 days, the cells were fixed with 4% paraformaldehyde and then stained with crystal violet at room temperature for 20 min. Colony numbers were calculated.

### EdU assay

Cells were seeded into coverslips that were placed in 24-well plates. EdU staining was performed with an EdU staining kit (C0071, Beyotime Institute of Biotechnology, Shanghai, China). Briefly, cells were fixed with 4% paraformaldehyde and then permeabilized with 0.5% Triton X-100. EdU solution was added to each well and incubated at 37 °C for 2 h.

### Wound healing assay

HCC cells were seeded into 6-well plates. When the cells reached 90% confluence, they were scratched with 200 µl pipette tips. After being washed with PBS, serum-free medium was used for further culture. Cells were obtained after 24 h of culture.

### Migration assay

Cells were resuspended in serum-free medium and seeded into the upper chambers of transwells. Medium containing 10% FBS was added to the lower chambers. The chambers were placed in 24-well plates and maintained at 37 °C for 24 h. Upper cells were removed, and migrated cells were stained with crystal violet.

### RT-qPCR analysis

A total of 1 ml TRIzol® (Thermo Fisher Scientific) was used to extract the total RNA of cells or liver tissues based on the manufacturer’s instructions. Then, cDNA was synthesized at 50 °C for 10 min and 85 °C for 5 s. SYBR Green (A46112, Thermo Fisher Scientific) was applied for qPCR according to the following conditions: initial denaturation at 94 °C for 30 s, followed by 35 cycles of denaturation at 94 °C for 5 s and extension at 60 °C for 30 s. The expression of the target genes was normalized to β-actin and determined by the 2^−∆∆Ct^ method.

### T cell-mediated killing assay

The peripheral blood mononuclear cells (PBMCs) were isolated from healthy donors and activated with 1 μg/ml coated anti-CD3 antibody (16–0037-81, Thermo Fisher Scientific) and 1 μg/ml soluble anti-CD28 antibody (14–0289-82, Thermo Fisher Scientific) for 48 h. The activated PBMCs were cocultured with HCC cell lines for 24 h at a ratio of 4:1.

### Flow cytometry

For CD133 and PD-L1 detection, cells were obtained and stained with anti-CD133 (17–1338-42, Thermo Fisher Scientific) or anti-PD-L1 antibodies (17–5983-42, Thermo Fisher Scientific) at 4 °C in the dark for 30 min. A LIVE/DEAD fixable violet dead cell stain kit (L34966, Thermo Fisher Scientific) was used to identify live cells.

For T cell detection, cells were treated with cell stimulation cocktail (plus protein transport inhibitors) (00–4975-93, Thermo Fisher Scientific) for 4 h before being harvested. Cells were then stained with anti-CD8 (17–0086-42, Thermo Fisher Scientific), anti-CD4 (12–0049-42, Thermo Fisher Scientific), or anti-CD56 (11–0566-42, Thermo Fisher Scientific) antibodies at 4 °C in the dark for 30 min. Cells were then fixed and permeabilized by intracellular Fixation & Permeabilization buffer set (88–8824-00, Thermo Fisher Scientific), subsequently incubated with anti-IFN-γ antibodies (47–7319-42, Thermo Fisher Scientific) at 4 °C in the dark for 30 min. A LIVE/DEAD fixable violet dead cell stain kit (L34966, Thermo Fisher Scientific) was used to identify live cells.

Apoptosis assays were performed using an Annexin V/PI apoptosis kit (70-AP101-100, MultiScience, Hangzhou, China). Cells were stained with Annexin V/PI solution at room temperature for 10 min. Then, the cells were acquired and analyzed using the BD FACSDiva program in a FACS LSRFortessa flow cytometer (BD Biosciences, San Jose, CA).

### Sphere formation assay

Cells were suspended in serum-free DMEM/F12 medium (11330032, Thermo Fisher Scientific) supplemented with 100 × N2 (17502001, Thermo Fisher Scientific, Inc.), 50 × B27 (17504044, Thermo Fisher Scientific), 20 ng/ml epidermal growth factor (EGF, AF-100–15, Peprotech, New Jersey, USA), 10 nmol fibroblast growth factor (FGF, 100-18B, Peprotech)), 5 μg/ml insulin (11376497001, Merck, St. Louis, USA), and 0.4% BSA. Then, the cells were seeded in low attachment 24-well plates and cultured for 7 days.

### Immunoprecipitation and western blot

Whole-cell lysates were extracted with cell lysis buffer for immunoprecipitation (IP) and western blot (P0013, Beyotime Biotechnology) and then quantified by BCA (23225, Thermo Fisher Scientific). For the immunoprecipitation assay, total proteins were incubated with 1 μg antibodies at 4 °C overnight, and protein A/G agarose (Santa Cruz Biotechnology, CA, USA) was added for another 2 h at 4 °C or incubated with anti-Flag magnetic beads at 4 °C overnight. The eluted immunoprecipitants were analyzed by SDS-PAGE.

For western blot analysis, protein samples were separated by SDS-PAGE and then transferred onto PVDF membranes, followed by blocking with 5% BSA for 1 h at room temperature. The membranes were then incubated with the indicated primary antibodies at 4 °C overnight. Next, membranes were stained with HRP-conjugated secondary antibody at room temperature for another 1 h. Antibodies used in this section were as follows: HERC2 (sc-515891, Santa Cruz), β-actin (66009–1-Ig, Proteintech, Chicago, IL, USA), phospho-JAK2 (Tyr1007/1008) (381556, Zenbio, Wuhan, China), CD133, (18470–1-AP, Proteintech), phospho-STAT3 (Tyr705) (381552, Zenbio), STAT3 (10253–2-AP, Proteintech), JAK2 (AF6022, Affinity), PTP1B (11334–1-AP, Proteintech), PD-L1 (66248–1-Ig, Proteintech), DYKDDDDK-tag (66008–4-Ig, Proteintech), HA-tag (3724, Cell Signaling Technology), GFP (66002–1-Ig, Proteintech), and ATP1A1 (14418–1-AP, Proteintech).

### Immunohistochemistry

Slides were hydrated, followed by an antigen retrieval procedure performed in citrate buffer (pH 6.0) at 100 °C for 10 min. Then, 3% H_2_O_2_ was used to block endogenous peroxidase activity at room temperature for 15 min. Slides were then blocked with goat serum at 37 °C for 1 h and stained with the indicated antibody at 4 °C overnight. Next, the slides were incubated with HRP-conjugated secondary antibody at 37 °C for 1 h. Determination of the immunoreactivity was conducted using an enhanced diaminobenzidine kit (TransGen Biotech, Beijing, China) and nuclear staining with hematoxylin.

### Immunofluorescence

For CD133 detection in Huh7 cells, the cells were fixed and permeabilized. After blocking with goat serum for 1 h, the cells were stained with CD133 (18470–1-AP, Proteintech) and HERC2 (sc-515891, Santa Cruz) primary antibodies overnight at 4 °C. The cells were then incubated with goat anti-mouse IgG H&L (Alexa Fluor® 488) (ab150113, Abcam, USA) and goat anti-rabbit IgG H&L (Alexa Fluor® 647) (ab150079, Abcam) antibodies for 1 h at 37 °C. The nucleus was stained with DAPI for 30 min at 37 °C. The images were obtained with a 100 × oil immersion objective on an Olympus FV1000 confocal microscope (Shinjuku, Tokyo, Japan).

For immunofluorescence analysis, HEK293T cells were transfected with HERC2-flag, PTP1B-mCherry or Jak2-GFP plasmids. To detect the interaction between HERC2 and PTP1B, cells were fixed and permeabilized. Next, the cells were incubated with an anti-DDDDK tag antibody (ab18230, Abcam) overnight at 4 °C. Nuclei were stained with DAPI for 30 min at 37 °C. To detect the interaction between PTP1B and JAK2, the cells were fixed and stained with DAPI for 30 min at 37 °C. The images were obtained with a 100 × oil immersion objective on an Olympus FV1000 confocal microscope (Shinjuku, Tokyo, Japan).

For total internal reflection fluorescence spectroscopy (TIRF) detection, HEK293T cells were cotransfected with PTP1B-mCherry and Sec61β-GFP plasmids. After being stimulated with 50 ng/ml IL-6, cells were immediately captured for 10 min alive.

### Statistical analysis

GraphPad Prism 8.0.1 was used for statistical analyses. The data are presented as the mean ± standard deviation (SD). Unpaired Student’s t test was used to compare the difference between two unpaired groups. Paired two-tailed Student’s t tests were applied for parametric data. Differences in the expression of HERC2 between tumors, adjacent tumors and HCC patients with different AFP levels were evaluated by the χ^2^ test. Kaplan–Meier and log-rank tests were used to determine survival rates. The correlations between HERC2 and stemness-related genes were assessed using Pearson’s test. *p* < 0.05 was considered to indicate a statistically significant difference. All experiments were independently repeated in triplicate.

## Results

### Upregulated HERC2 is associated with inflammation-related HCC progression

**Fig. 1:**
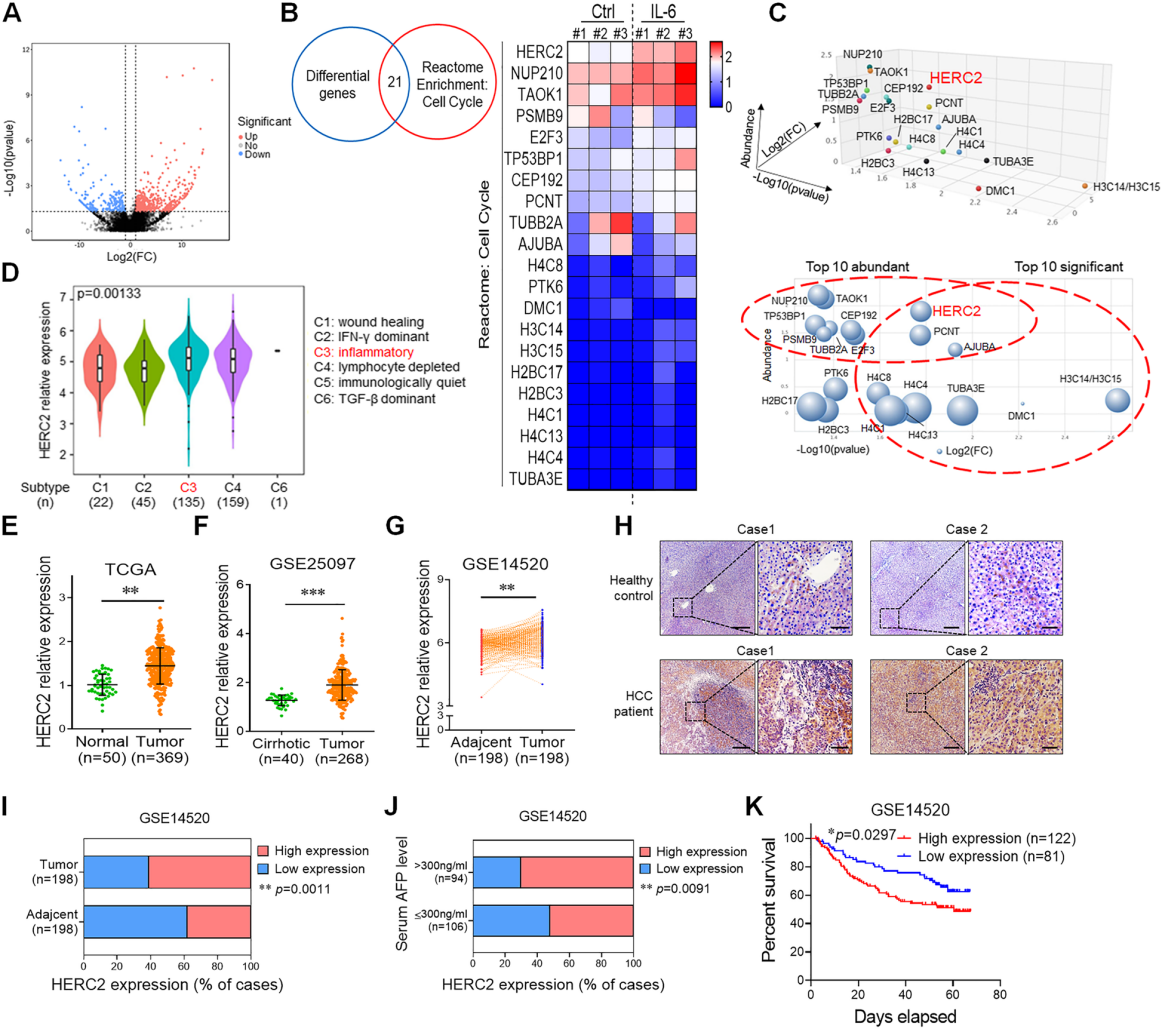
Upregulated HERC2 was associated with the progression and poor prognosis of HCC. **A-C** Isolated human hepatocytes were treated with 50 ng/ml IL-6 for 20 min, followed by RNA-sequencing analysis. **A** Volcano blot displaying twofold change differential genes post IL-6 treatment. **B** Venn diagram exhibiting common differential genes between twofold change differential genes post IL-6 treatment and genes involved in the cell cycle pathway based on reactome enrichment. Heatmap displayed the expression of common differentially expressed genes in human primary hepatocytes. **C** 3-D dot plot and bubble diagram displaying significance, fold change, and abundance of indicated genes. **D** Violin plot showing the HERC2 expression in tumor tissues with different immune subtypes based on TCGA dataset (*n* = 362). **E** Expression levels of HERC2 in normal livers (*n* = 50) and HCC livers (*n* = 369) based on TCGA datasets. **F** Expression levels of HERC2 in cirrhotic livers (*n* = 40) and HCC livers (*n* = 268) in the GSE25097 dataset. **G** Expression levels of HERC2 in the tumor area and adjacent nontumor area (*n* = 198) in HCC patients according to GSE14520 datasets. **H** Immunohistochemical analysis of HERC2 expression in healthy livers and HCC livers, scale bars (left) = 100 μm, scale bars (right) = 25 μm. **I** Proportion of high HERC2 expression and low HERC2 expression in tumor or non-tumor liver tissues (*n* = 198) based on GSE14520 datasets. **J** Proportion of high HERC2 expression and low HERC2 expression in HCC patients with low serum AFP levels (*n* = 106) or high AFP levels (*n* = 94) based on GSE14520 datasets. (K) Cox regression analysis of HCC patients with high HERC2 expression (*n* = 122) and low HERC2 expression (*n* = 81) based on GSE14520 datasets. **p < 0.01, ***p < 0.001. Data from one representative experiment of three independent experiments are presented

Among the multiple cytokines, the role of IL-6 in the development and progression of inflammation-associated HCC has been widely described [4, 6]. We found elevated IL-6 levels in liver tissues from HCC patients with multinodular compared to those without multinodular (Supplementary Fig. S1A). Besides, patients with high predicted risk metastasis signature also displayed higher IL-6 expression than patients with low predicted risk metastasis signature, which indicated the oncogenic role of IL-6 in inflammation-associated HCC (Supplementary Fig. S1B). To explore the critical molecules involved in inflammation-mediated liver tumorigenesis, hepatocytes isolated from human livers were stimulated with inflammatory cytokine IL-6, followed by RNA-Seq analysis. twofold change was set as the threshold and the results showed a significant difference between samples with or without inflammatory stimulation. Upon IL-6 stimulation, a total of 732 and 235 genes were upregulated and downregulated, respectively (Fig. 1A). Given that inflammatory condition strongly regulates the cell cycle process during tumorigenesis, we determined 21 differential genes involved in the cell cycle pathway, and heatmap illustrated the distribution and levels of the genes in cells with or without inflammatory stimulation (Fig. 1B). We then constructed a 3-D model to analyze the significance, abundance and fold change of the differential transcripts at the same time. Among all the transcripts, HERC2 was identified for further investigation (Fig. 1C). RT-qPCR analysis (Supplementary Fig. S1C) and western blot assay (Supplementary Fig. S1D) were then performed to confirm that IL-6-induced HERC2 expression in hepatocytes. Notably, the high expression of HERC2 in tumor tissues was related to inflammatory immune subtype (Fig. 1D), which indicated a potential role of HERC2 in inflammation-related liver tumorigenesis. To further investigate the role of HERC2 in inflammation-related HCC progression, the expression profile of HERC2 was analyzed based on HCC public datasets. We found elevated HERC2 expression in tumor tissues compared to the normal tissues (Fig. 1E). The preneoplastic setting of the cirrhotic background provides a conducive environment for HCC development [19]. Interestingly, increased HERC2 expression was also observed in HCC liver tissues compared to cirrhotic liver tissues (Fig. 1F). Similarly, tumor tissues exhibited higher HERC2 levels than the matched adjacent tissues in HCC patients (Fig. 1G). Immunohistochemistry analysis validated that liver tissues obtained from HCC patients displayed enhanced HERC2 expression compared to the normal liver tissues (Fig. 1H). Furthermore, we categorized HERC2 levels into high and low expression in HCC patients, and found that a greater frequency of high HERC2 expression was exhibited in HCC tissues than in the adjacent tissues (Fig. 1I). Consistently, liver tissues obtained from HCC patients with elevated serum alpha-fetoprotein (AFP) levels exhibited a more HERC2 high expression profile than those from HCC patients with low serum AFP levels (Fig. 1J). In addition, Cox regression analysis suggested that HERC2 expression was identified as an independent prognostic factor for HCC patients with cirrhosis, and high HERC2 expression led to a poor prognosis (Fig. 1K and Supplementary Fig. S1E). Accordingly, these data suggested that inflammatory stimulation induced HERC2 expression in hepatocytes and that upregulated HERC2 was associated with the progression and poor prognosis of HCC.

### HERC2 promotes the malignant phenotype of HCC cells

**Fig. 2:**
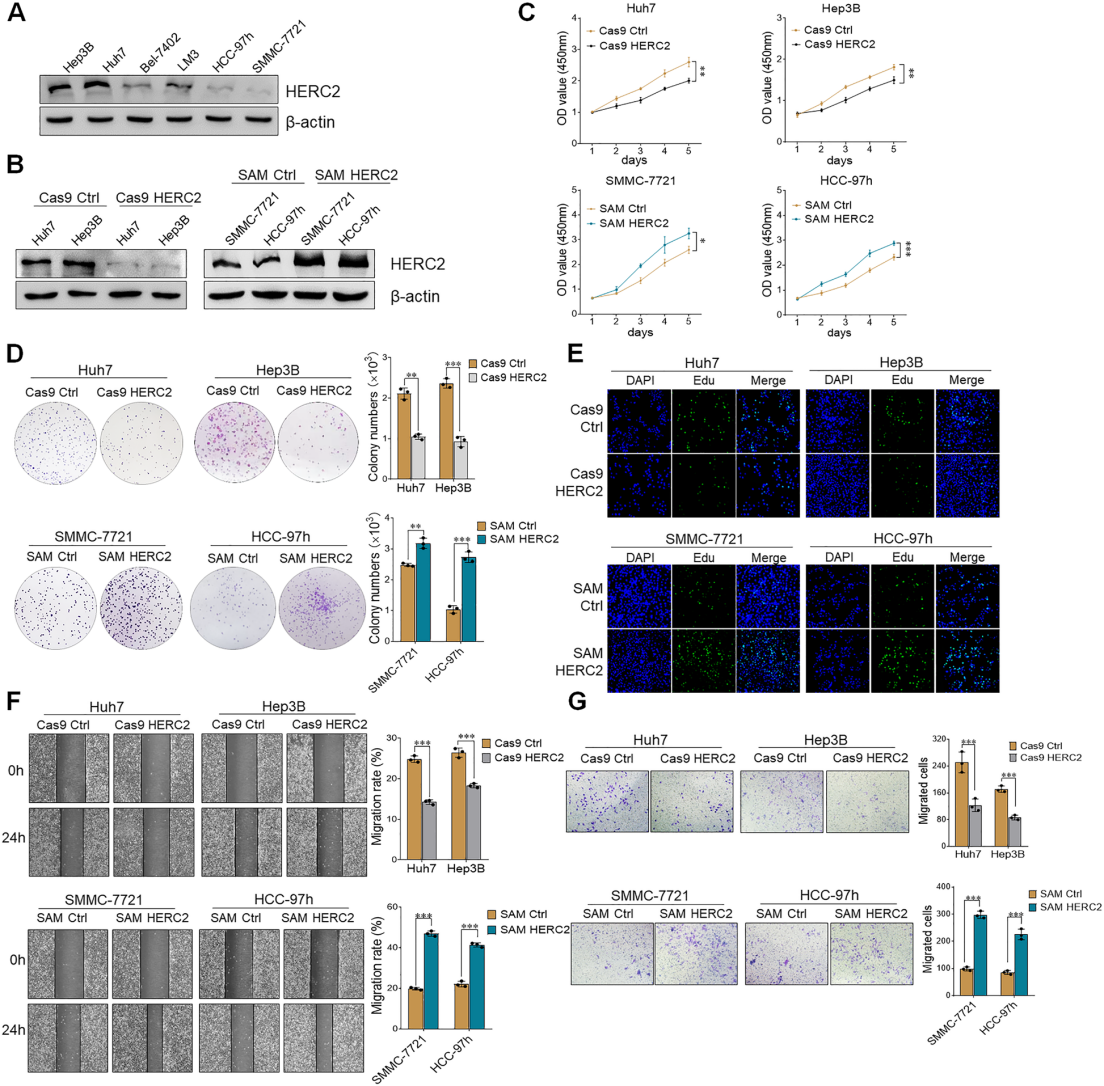
HERC2 promoted the malignant phenotype of HCC cells. **A** Expression levels of HERC2 in Hep3B, Huh7, Bel-7402, LM3, HCC-97 h, and SMMC-7721 cell lines were measured by immunoblotting assay. **B** Western blot analysis of HERC2 protein levels both in HERC2 knockout and overexpression cell lines. **C** A CCK-8 assay was used to detect cell proliferation in both HERC2 knockout and HERC2 overexpression cell lines. **D** A colony formation assay was performed to investigate cell proliferation in HERC2 knockout and overexpression cell lines. **E** Immunofluorescent detection of EdU was employed to determine the proliferation of HERC2 knockout and overexpression cell lines. Wound healing assays **F** and migration tests **G** were used to detect the migration ability of HERC2 knockout and overexpression HCC cells. *p < 0.05, **p < 0.01, ***p < 0.001. Data from one representative experiment of three independent experiments are presented

The HERC2 expression profile of different HCC cell lines by means of immunoblotting assay showed that Huh7 and Hep3B cells exhibited a high level of HERC2 while limited HERC2 expression was presented in SMMC-7721 and HCC-97 h cells (Fig. 2A). Thus, knockout of HERC2 expression was conducted in Huh7 and Hep3B cells by the CRISPR-Cas9 system. Meanwhile, HERC2 overexpression was established in SMMC-7721 and HCC-97 h cells (Fig. 2B). As indicated by CCK-8 and colony formation assays, the knockout of HERC2 significantly attenuated the proliferation ability of HCC cells, while overexpression of HERC2 promoted HCC cell proliferation (Fig. 2C and D). Similarly, the EdU assay demonstrated that HERC2 deficiency inhibited DNA synthesis in HCC cells, while overexpression of HERC2 boosted HCC cell DNA synthesis (Fig. 2E). We then tried to clarify the effect of HERC2 on HCC metastasis in vitro. Wound healing assays showed that HERC2 knockout reduced the wound-healing efficacy of Huh7 and Hep3B cells. In contrast, accelerated cell migration was observed in HERC2-overexpressing SMMC-7721 and HCC-97 h cells (Fig. 2F). Furthermore, migration assays showed decreased cell counts of HERC2 knockout Huh7 and Hep3B HCC cells in the lower chamber compared to their counterparts, while more migrated cells were found in the lower chamber than in the control groups when HERC2 levels were overexpressed in SMMC-7721 and HCC-97 h cells (Fig. 2G). Altogether, these results indicated that HERC2 promoted the malignant phenotype of HCC cells.

### HERC2 enhances the stemness of HCC cells

**Fig. 3:**
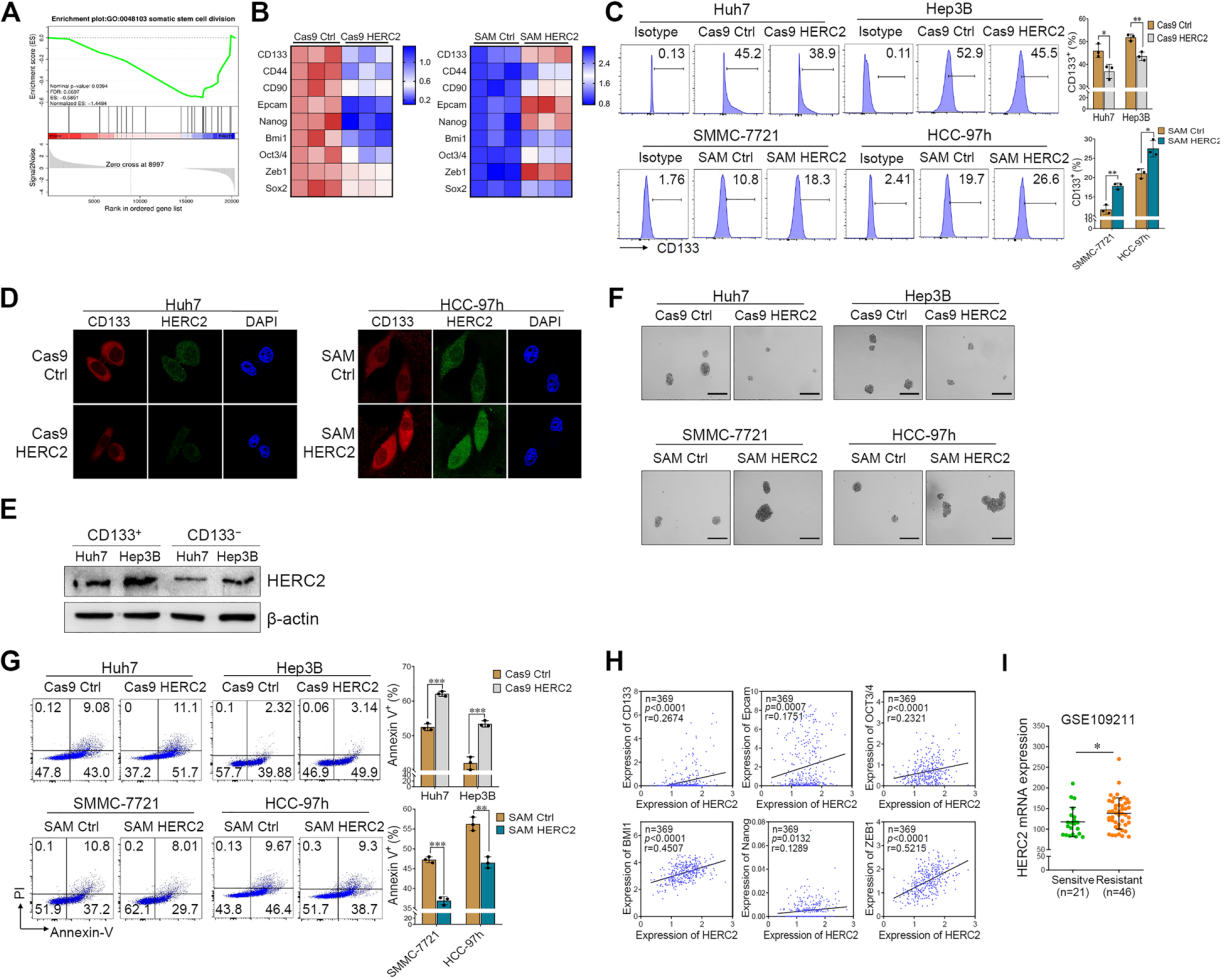
HERC2 promoted the stemness of HCC cells. (**A**) HERC2 knockout Huh7 cells were treated with 50 ng/ml IL-6 for 24 h and then subjected to RNA-seq analysis. GSEA revealed enrichment of the somatic stem cell division-related gene signature in HERC2 knockout cells. **B-D** HERC2 knockout and overexpression cells were treated with 50 ng/ml IL-6 for 24 h. **B** RT-qPCR analysis was used to evaluate the mRNA levels of cancer stem cell-related genes. **C** Flow cytometry analysis was used to determine CD133 expression. **D** An immunofluorescence assay was conducted for CD133 detection. **E** CD133-positive and -negative cells were isolated from the Huh7 and Hep3B cell lines, respectively, through magnetic cell sorting. Western blot analysis was performed to detect HERC2 levels. (**F**) Cells were cultured under 100 × N2, 50 × B27, 20 ng/ml EGF, 10 nmol FGF, 5 μg/ml insulin, and 0.4% BSA conditions for 7 days, scale bars = 100 μm. **G** Cells were treated with 20 μM sorafenib for 24 h. A flow cytometry assay was used to determine the percentage of apoptotic cells. **H** The correlation between HERC2 expression and levels of cancer stem cell-related genes (e.g., CD133, CD44, CD90, Epcam, OCT3/4, Bmi1, Nanog, and Zeb1) based on TCGA datasets (*n* = 369). **I** The levels of HERC2 in liver tissues from sorafenib-sensitive and -resistant patients were analyzed according to GSE109211 datasets. *p < 0.05, **p < 0.01, ***p < 0.001. Data from one representative experiment of three independent experiments are presented

CSCs promote HCC progression, metastasis, and immune evasion [9]. Treatment with IL-6 significantly induced CD133 expression in HCC cells [20]. Herein, the “stem cell division pathway” was differentially identified in HERC2 knockout Huh7 cells and their counterparts upon inflammatory stimulation based on GSEA enrichment analysis (Fig. 3A). Therefore, we raised the question of whether HERC2 regulates the stemness of HCC cells. Indeed, RT-qPCR assay demonstrated that stemness-related gene levels (e.g., CD133, Epcam, Nanog, Bmi1, OCT3/4, Zeb1, and Sox2) were decreased in HERC2 knockout Huh7 cells under inflammatory stimulation, while HERC2 overexpression promoted the expression of these genes (Fig. 3B). Since CD133 is a generally known CSC marker in HCC, CD133 expression was then evaluated by flow cytometry and immunofluorescence. As expected, attenuated expression of CD133 was detected in HERC2 knockout cells, and the CD133 level was significantly increased when HERC2 was overexpressed (Fig. 3C and D). CD133^+^ and CD133^−^ cells were further isolated from Huh7 and Hep3B cells to clarify the correlation between HERC2 expression and CD133 levels, and we observed higher HERC2 levels in CD133^+^ cells than those in CD133^−^ cells (Fig. 3E). Self-renewal ability is one of the typical stemness-related properties. Thus, a sphere formation assay was performed. Limited sphere formation ability was observed in hepatocytes when HERC2 expression was ablated, while increased diameter of spheres was exhibited in HERC2-overexpressing cells (Fig. 3F). Resistance to chemotherapeutic drugs is another characteristic of CSCs. We then investigated whether HERC2 regulated the drug-resistance ability of HCC cells. Sorafenib, the most common chemotherapeutic agent for HCC therapy, was chosen to induce HCC cell apoptosis. Annexin V-PI staining demonstrated that HERC2 deficiency attenuated the drug-resistance ability of Huh7 and Hep3B cells. In contrast, HERC2 overexpression increased the viability of SMMC-7721 and HCC-97 h cells during sorafenib treatment (Fig. 3G). To further confirm the role of HERC2 in stemness-related features, a correlation between HERC2 levels and CSC markers in tissues from HCC patients was analyzed. We found that HERC2 levels were positively related to various stemness-related markers in the liver tissues of HCC patients (Fig. 3H). In addition, liver tissues from sorafenib-resistant patients exhibited higher HERC2 levels than those from sorafenib-sensitive patients (Fig. 3I).

### HERC2 -mediated PD-L1 expression promotes immune evasion of HCC cells

**Fig. 4:**
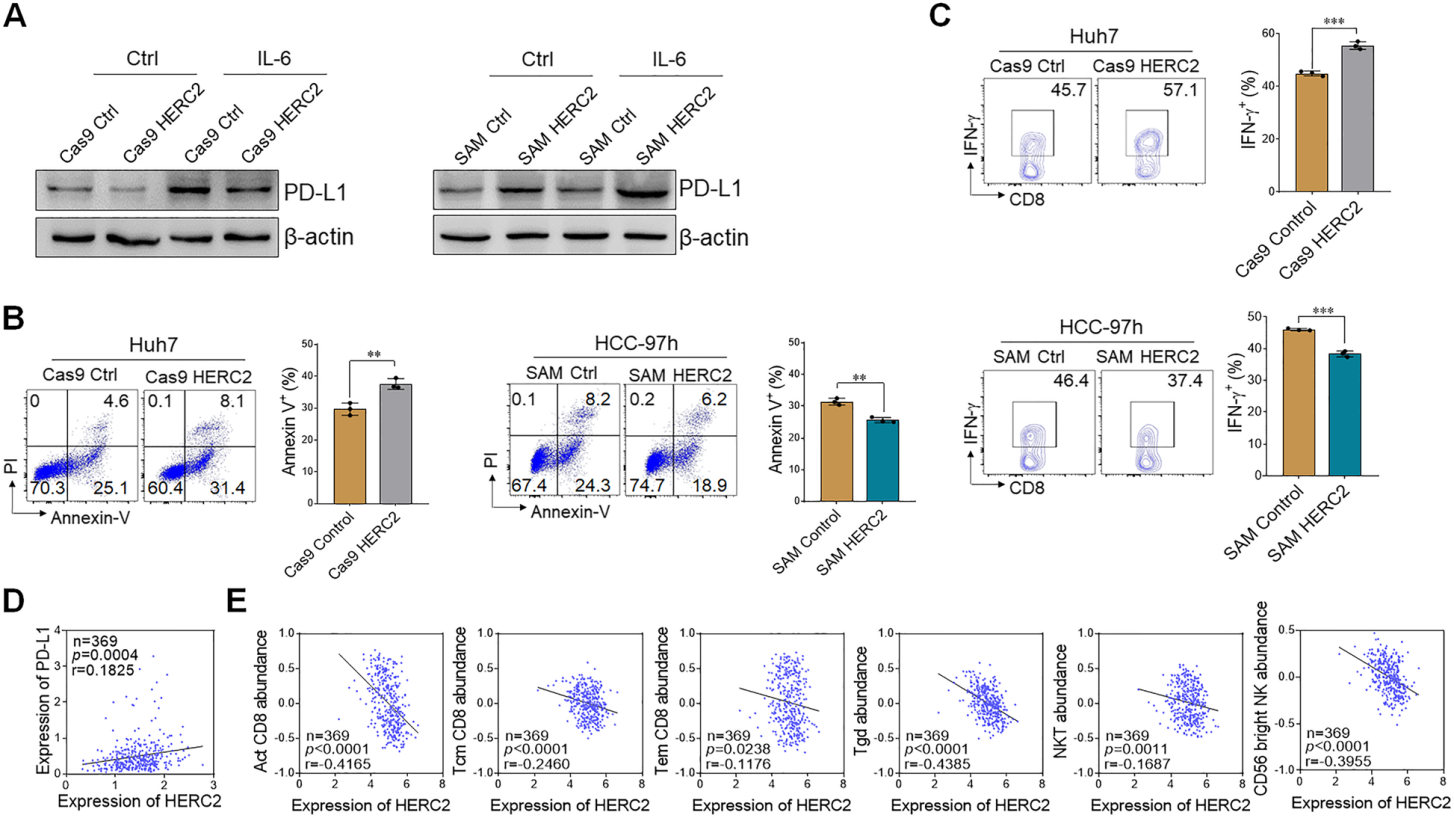
HERC2 participated in the immune evasion of HCC cells. **A** HERC2-deficient Huh7 cells and HERC2-overexpressing HCC-97 h cells were treated with 50 ng/ml IL-6 for 24 h and then subjected to western blot assay for PD-L1 detection. **B** and **C** Activated PBMCs were cocultured with Huh7 cells or HERC2-overexpressing HCC-97 h cells at the ratio of 4:1 for 24 h. Apoptosis of HCC cells was detected by flow cytometry assay (**B**). **C** IFN-γ levels of CD8^+^ T cells were determined by flow cytometry analysis. **D** The correlation between HERC2 expression and levels of PD-L1 based on TCGA datasets (*n* = 369). **E** The correlation between HERC2 expression and liver immune cell recruitment based on TCGA datasets (*n* = 369). **p < 0.01, ***p < 0.001. Data from one representative experiment of three independent experiments are presented

Tumor cells expressed PD-L1 coupled with PD-1 on activating T cells, resulting in the apoptosis and dysfunction of T cells, which has been documented as a critical procedure in tumor cell-mediated immune evasion to sustain the tumorigenic process [21]. CSCs were found to be more resistant to T cell-mediated cytotoxicity than non-CSCs, due to the enrichment of PD-L1 [22]. We thus investigated whether HERC2 regulates anti-tumor T cell immunity via modulating PD-L1 expression in HCC cells. The result showed that HERC2 knockout reduced PD-L1 expression in HCC cells, while HERC2 overexpression significantly increased the PD-L1 level in HCC cells (Fig. 4A and Supplementary Fig. S2A). T cell-mediated killing assay was then performed in vitro by coculturing activated PBMCs from healthy donors with HCC cell lines. We observed that HERC2-deficient HCC cells were more vulnerable to T cell-mediated cytotoxicity. By contrast, more resistant HCC cells were against T cell killing when HERC2 was overexpressed (Fig. 4B). Additionally, the IFN-γ levels of CD8^+^ T cells, CD4^+^ T cells, and CD56^+^ NK cells were determined by flow cytometry. HERC2-deficient HCC cells promoted CD8^+^ T cells, CD4^+^ T cells, and CD56^+^ NK cells to produce IFN-γ, while HERC2-overexpressed HCC cells limited IFN-γ secretion from CD8^+^ T cells, CD4^+^ T cells, and CD56^+^ NK cells (Fig. 4C and Supplementary Fig. S2B and S2C). To further confirm our results in clinical samples, the relationship between HERC2 expression and the immune microenvironment in HCC was analyzed based on TCGA datasets. We found that HERC2 expression was positively correlated to PD-L1 levels in the liver tissues of HCC patients (Fig. 4D). Furthermore, hepatic HERC2 expression was negatively correlated to the abundance of activated CD8^+^ T cell, central memory CD8^+^ T cell, effector memory CD8^+^ T cell, γδT cell, NKT cell, and CD56^bright^ NK cell, which have been reported to express PD-1 (Fig. 4E). Taken together, our data indicated that HERC2 promotes the immune evasion of HCC cells.

### HERC2 controls stemness and immune evasion of HCC cells through JAK2/STAT3 signaling

**Fig. 5:**
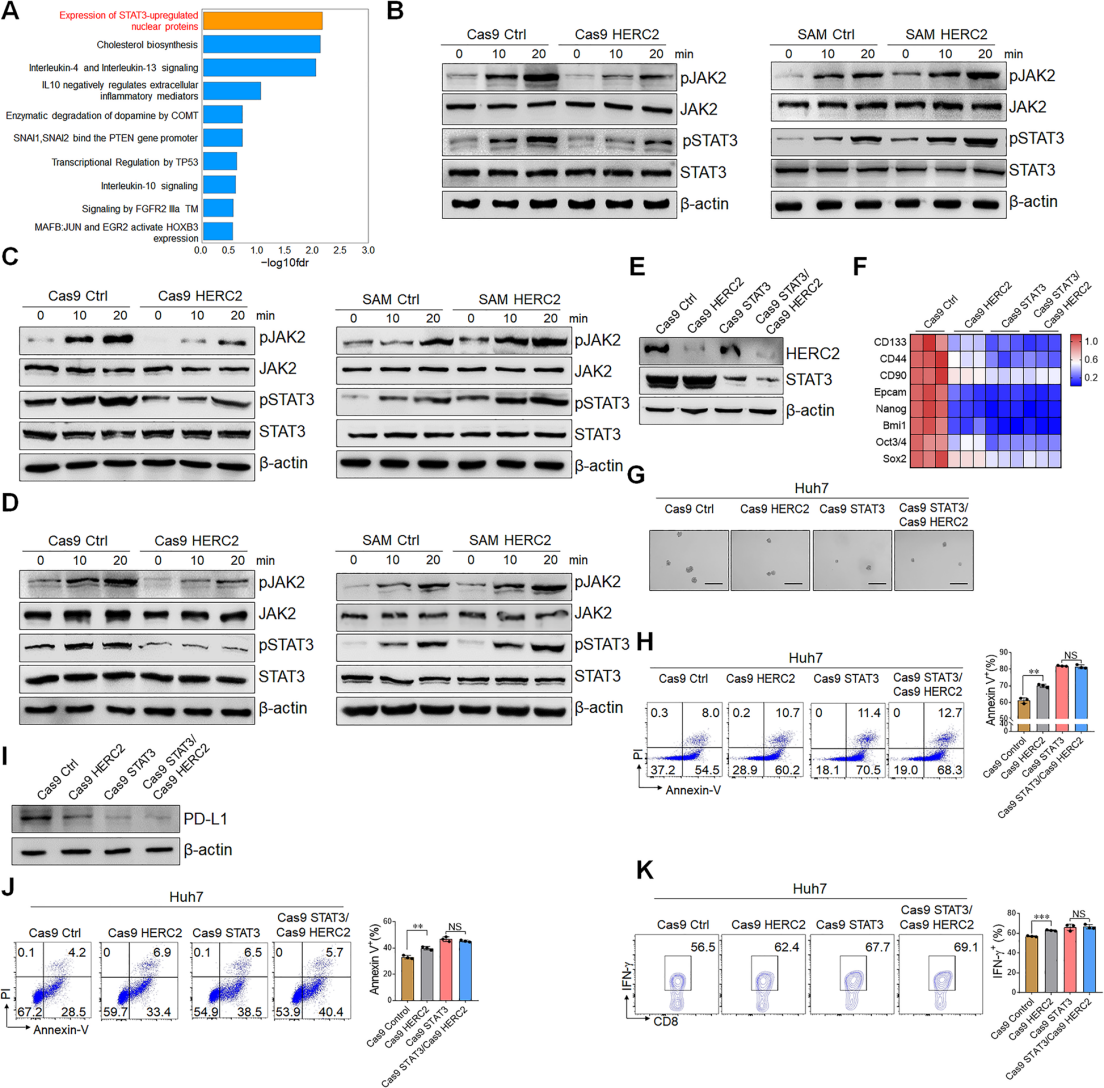
HERC2 promoted the stemness and immune evasion of HCC cells through JAK2/STAT3 signaling. **A** HERC2 knockout Huh7 cells were treated with 50 ng/ml IL-6 for 24 h and then subjected to RNA-seq analysis. Reactome pathway analysis displayed the most enriched pathways. **B-D** HERC2-deficient Huh7 cells and HERC2-overexpressing HCC-97 h cells were treated with 50 ng/ml IL-6 (**B**), 50 ng/ml IL-11 (**C**), or 20 ng/ml EGF (**D**), respectively. The phosphorylation of JAK2 and STAT3 was determined by western blot analysis. **E** HERC2 and STAT3 double-deficient Huh7 cell lines were established. **F** The cells were treated with 50 ng/ml IL-6 for 24 h. The RT-qPCR assay was used to detect the mRNA expression of cancer stem cell-related genes. **G** The cells were cultured in a conditioned medium with 100 × N2, 50 × B27, 20 ng/ml EGF, 10 nmol FGF, 5 μg/ml insulin, and 0.4% BSA for 7 days, scale bars = 100 μm. **H** The cells were treated with 20 μM sorafenib for 24 h. A flow cytometry assay was used to determine the percentage of apoptotic cells. **I** Cells were treated with 50 ng/ml IL-6 for 24 h and then subjected to western blot assay for PD-L1 detection. **J** and **K** Activated PBMCs were cocultured with HCC cells at the ratio of 4:1 for 24 h. Apoptosis of HCC cells was detected by flow cytometry assay (**J**). **K** IFN-γ levels of CD8^+^ T cells were determined by flow cytometry analysis. NS: not significant, **p < 0.01, ***p < 0.001. Data from one representative experiment of three independent experiments are presented

To characterize the underlying mechanism of HERC2 in HCC cell regulation, Reactome pathway enrichment was performed among HERC2-deficient Huh7 cells and control cells upon inflammatory stimulation. Strikingly, the “expression of STAT3-upregulated nuclear proteins” pathway exhibited the most significant difference (Fig. 5A), which indicated that HERC2 might be involved in the process of STAT3 phosphorylation. We then tried to validate whether HERC2 modulates the activation of the JAK2/STAT3 signaling pathway in hepatocytes stimulated with IL-6. As expected, HERC2 deficiency significantly inhibited STAT3 phosphorylated levels, while HERC2 overexpression promoted STAT3 phosphorylation (Fig. 5B). In addition, IL-11, another IL-6 family cytokine, and EGF, which activates JAK2/STAT3 signaling in a gp130-independent way, were used to induce JAK2/STAT3 signaling activation. We found HERC2 also affected IL-11 or EGF-induced STAT3 phosphorylation (Fig. 5C and D). Additionally, the single cell RNA-seq data of HCC specimens from GSE146115 datasets were analyzed. The tumor cells were divided into HERC2-positive and HERC2-negative groups based on the HERC2 expression (Supplementary Fig. S3A and B). Notably, HERC2-positive tumor cells expressed higher IL-6/JAK/STAT3-related genes and STAT3-targeted genes than HERC2-negative tumor cells, which also indicated the regulatory role of HERC2 in JAK2/STAT3 signaling (Supplementary Fig. S3C and D). Together, these results suggested that HERC2 promoted the STAT3 activation induced by either gp130-dependent or independent pathways. To further validate whether HERC2 regulated HCC cells through STAT3 signaling, STAT3-deficient/HERC2 knockout Huh7 and STAT3-deficient/HERC2 overexpressing HCC-97 h cells were established by the CRISPR-Casp9 system (Fig. 5E and Supplementary Fig. S4A). We observed that STAT3 deficiency abolished the promotive effect of HERC2 on HCC cell proliferation (Supplementary Fig. S4B and C). Similarly, when STAT3 expression was ablated, neither knockout nor overexpression of HERC2 affected the HCC cell migration (Supplementary Fig. S4D and E). We then investigated whether HERC2 regulated the stemness of HCC cells through STAT3 signaling. We found HERC2 exhibited limited influence on stemness-related gene expression in STAT3 knockout conditions (Fig. 5F and Supplementary Fig. S5A). Consistently, the effect of HERC2 on sphere formation was abolished in STAT3 knockout HCC cells (Fig. 5G and Supplementary Fig. S5B). Both HERC2 knockout and overexpression cells showed comparable cell viability to controls under sorafenib exposure when STAT3 expression was knocked out (Fig. 5H and Supplementary Fig. S5C). Moreover, STAT3 ablation dismissed the effect of HERC2 on PD-L1 expression in HCC cells (Fig. 5I and Supplementary Fig. S5D and E). Comparable viability was also found between HERC2 knockdown or overexpressed HCC cells and counterparts in the condition of coculturing with PBMC when STAT3 was knockdown (Fig. 5J and Supplementary Fig. S5F). Consistently, neither HERC2 knockout nor overexpression in HCC cells affected IFN-γ production from CD8^+^ T cells, CD4^+^ T cells, and CD56^+^ NK cells when STAT3 expression was ablated (Fig. 5K and Supplementary Fig. S5G-I). Overall, we concluded that HERC2 enhanced the malignancy, stemness, and immune evasion of HCC cells through JAK2/STAT3 signaling.

### HERC2 modulates JAK2/STAT3 signaling through interaction with PTP1B

**Fig. 6:**
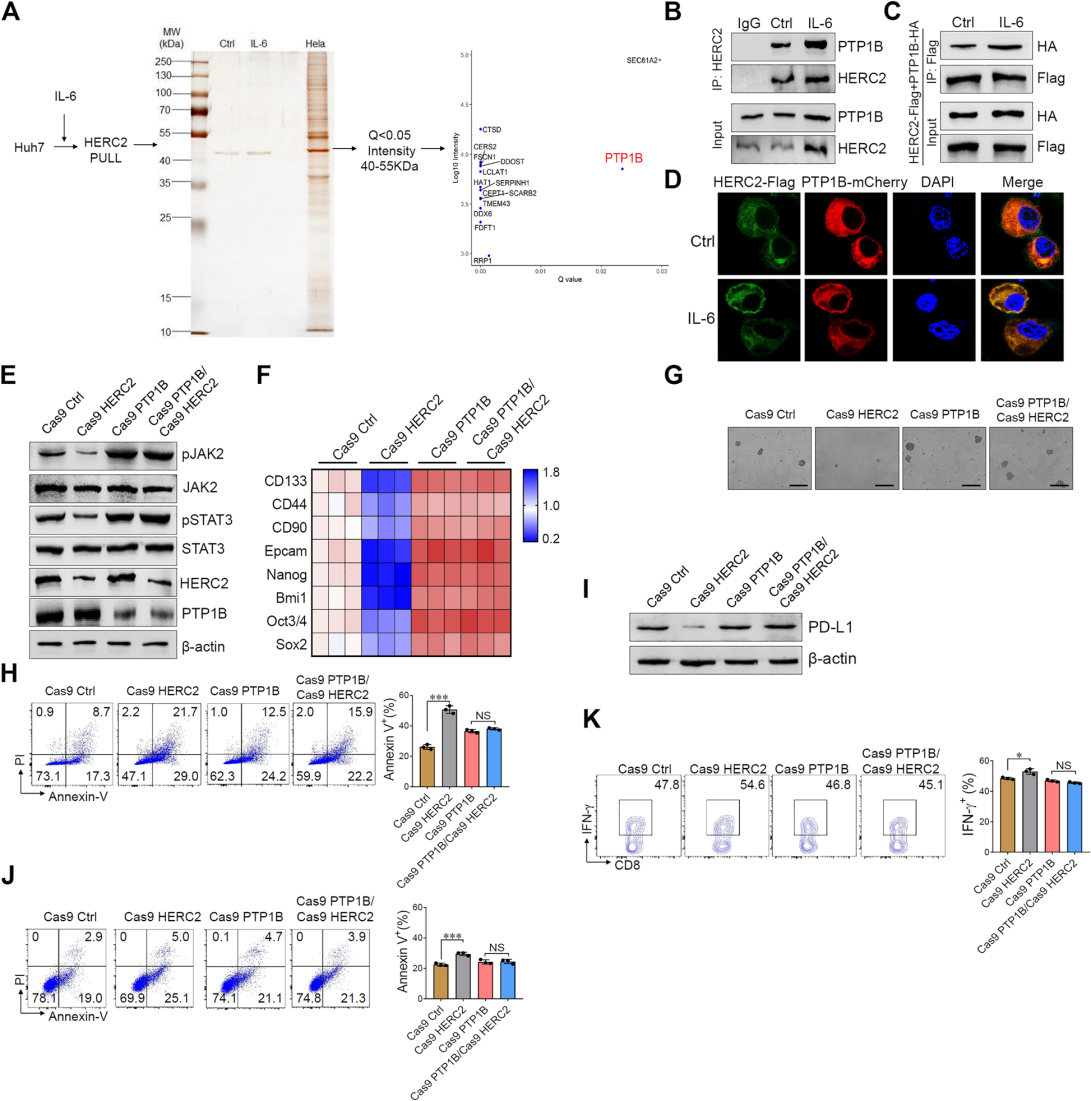
HERC2 regulated JAK2/STAT3 signaling through direct interaction with PTP1B. **A** Huh7 cells were treated with 50 ng/ml IL-6 for 20 min. The extracted protein was precipitated with an antibody against HERC2 and separated by SDS-PAGE followed by silver staining. On the other hand, the band was analyzed with 4-D label-free quantitative proteomics. **B** Huh7 cells were treated with 50 ng/ml IL-6 for 20 min, and the interaction between HERC2 and PTP1B was validated by an immunoprecipitation assay. **C-D** HEK293T cells were co-transfected with HERC2-Flag and PTP1B-HA (**C**) or PTP1B-mcherry (**D**) plasmids. **C** Immunoprecipitation was performed using an anti-FLAG magnetic beads. The presence of coprecipitated HERC2 was determined by immunoblotting with the anti-HA antibody. **D** The colocalization of HERC2 and PTP1B in the cells was analyzed by immunofluorescence assay. **E** HERC2 and PTP1B double-deficient Huh7 cell lines were established. The cells were treated with 50 ng/ml IL-6 for 20 min, and the phosphorylation levels of JAK2 and STAT3 were detected by western blot analysis. **F** Cells were treated with 50 ng/ml IL-6 for 24 h. The RT-qPCR assay was used to detect the mRNA expression of CSC-related genes. **G** Cells were cultured with medium containing 100 × N2, 50 × B27, 20 ng/ml EGF, 10 nmol FGF, 5 μg/ml insulin, and 0.4% BSA for 7 days, scale bars = 100 μm. **H** The cells were treated with 20 μM sorafenib for 24 h. A flow cytometry assay was used to determine the percentage of apoptotic cells. **I** Cells were treated with 50 ng/ml IL-6 for 24 h and then subjected to western blot assay for PD-L1 detection. **J** and **K** Activated PBMCs were cocultured with HCC cells at the ratio of 4:1 for 24 h. Apoptosis of HCC cells was detected by flow cytometry assay (**J**). **K** IFN-γ levels of CD8^+^ T cells were determined by flow cytometry analysis. NS: not significant, *p < 0.05, **p < 0.01, ***p < 0.001. Data from one representative experiment of three independent experiments are presented

Next, we investigated the molecular mechanism by which HERC2 promoted JAK2/STAT3 signal activation. The HERC2 immunoprecipitation complex was pulled down to identify potential HERC2-interacting proteins. The immunoprecipitants were separated by SDS-PAGE followed by silver staining. We found a protein band at 40–55 kDa, and among the recognized proteins. The band was extracted from the gel and subjected to mass spectrometry analysis (Fig. 6A). Among the candidates, PTP1B has been widely reported to interact with JAK2 and inhibit JAK2 signaling through dephosphorylation of JAK2 [23–25]. We confirmed the interaction between HERC2 and PTP1B in Huh7 cells. Interestingly, IL-6 stimulation significantly enhanced the interaction between HERC2 and PTP1B (Fig. 6B). To further identify the direct interaction between HERC2 and PTP1B, HERC2 and PTP1B plasmids were co-transfected into HEK293T cells. Both immunoprecipitation assays (Fig. 6C) and immunofluorescence analysis (Fig. 6D) revealed a direct interaction between HERC2 and PTP1B. Moreover, PTP1B knockout Huh7 and HCC-97 h cells were established by the CRISPR-Cas9 system. Strikingly, PTP1B knockout significantly abolished the effect of HERC2 knockout or overexpression on JAK2/STAT3 signal activation (Fig. 6E and Supplementary Fig. S6A). We further demonstrated that HERC2 exhibited limited influence on HCC cell proliferation (Supplementary Fig. S6B and C) and migration (Supplementary Fig. S6D) when PTP1B was knocked out. In addition, the effect of HERC2 on stemness-related gene expression (Fig. 6F and Supplementary Fig. S7A), sphere formation ability (Fig. 6G and Supplementary Fig. S7B), and sorafenib resistance (Fig. 6H and Supplementary Fig. S7C) were abolished in PTP1B knockout HCC cells. Moreover, ablation of PTP1B expression led to the restricted effect of HERC2 on PD-L1 expression (Fig. 6I and Supplementary Fig. S7D and E) and immune evasion in HCC cells (Fig. 6J and K and Supplementary Fig. S7F-I). Overall, these data indicated that HERC2 directly interacted with PTP1B and modulated JAK2/STAT3 signaling through PTP1B.

### HERC2 inhibited PTP1B translocation to ER-PM junctions, thereby limiting the interaction between PTP1B and JAK2

**Fig. 7:**
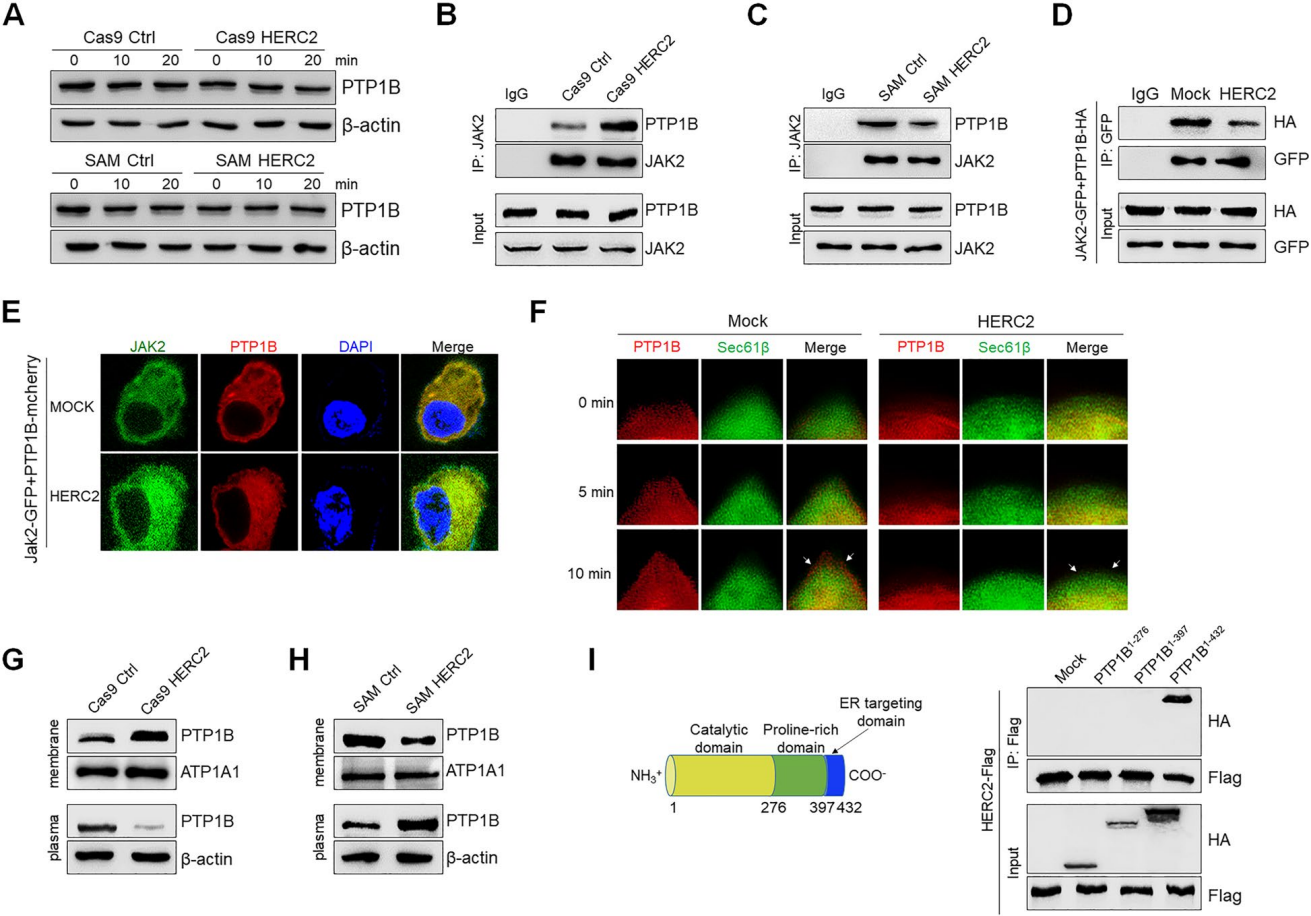
HERC2 inhibited PTP1B translocation in ER-PM junction. **A** HCC cells were treated with 50 ng/ml IL-6 for the indicated time points, and then the expression level of PTP1B was detected with western blot analysis. HERC2 knockout (**B**) and HERC2 overexpression (**C**) cells were treated with 50 ng/ml IL-6 for 20 min, and an immunoprecipitation assay was performed to analyze the interaction between JAK2 and PTP1B. **D-E** HEK293T cells were co-transfected with JAK2-GFP and PTP1B-HA (**D**) or PTP1B-mCherry (**E**) plasmids after being transfected with HERC2 or mock plasmids. The cells were then treated with 50 ng/ml IL-6 for 20 min. **D** An immunoprecipitation assay was performed to evaluate the interaction between JAK2 and PTP1B. **E** An immunofluorescence assay was performed to assess the colocalization of JAK2 with PTP1B. **F** HEK293T cells were co-transfected with PTP1B-mCherry and Sec61β-GFP plasmids. After transfection with HERC2 or mock plasmids, the cells were treated with 50 ng/ml IL-6 for 10 min. The localization of the indicated proteins was observed by total internal reflection fluorescence spectroscopy (TIRF). **G-H** HERC2 knockout (**G**) and HERC2 overexpression (**H**) cells were treated with 50 ng/ml IL-6 for 20 min, then membrane protein was isolated and tested for PTP1B levels by western blot analysis. **I** HEK293T cells were co-transfected with HERC2-Flag and PTP1B mutant-HA plasmids, an immunoprecipitation assay was performed to determine which domain of PTP1B that interacted with HERC2. Data from one representative experiment of three independent experiments are presented

HERC2 has been widely reported as an E3 ubiquitin ligase [26, 27]. We thus first evaluated whether HERC2 participated in proteasome-mediated PTP1B degradation. Strikingly, no significant difference in the protein level of PTP1B was observed under HERC2 knockout or HERC2 overexpression conditions (Fig. 7A). Notably, HERC2 has also been identified to function as a scaffolding factor that supports the protein–protein complex formation and mediates intracellular protein translocation [28]. We then investigated whether HERC2 affected the crosstalk between PTP1B and JAK2. Interestingly, HERC2 knockout promoted PTP1B interaction with JAK2, while HERC2 overexpression limited the PTP1B-JAK2 association in HCC cells (Fig. 7B and C). To confirm the results, HEK293T cells were co-transfected with PTP1B and JAK2 plasmids. Both immunoprecipitation assay (Fig. 7D) and immunofluorescence analysis (Fig. 7E) demonstrated that HERC2 suppressed the interaction between PTP1B and JAK2. It should be noted that PTP1B has been identified as an ER-anchored protein. Emerging evidence indicates that ER-plasma membrane (PM) junctions are membrane microdomains essential for communication between the ER and the PM [29, 30]. Most importantly, the results showed that HERC2 overexpression inhibited the translocation of PTP1B from the ER to ER-PM junctions (Fig. 7F and Supplementary Video). Furthermore, plasma membrane proteins were isolated from HERC2 knockout and HERC2-overexpressing HCC cells. We observed increased PTP1B levels in PM isolated from HERC2 knockout HCC cells compared to that from control cells. In contrast, decreased PTP1B levels were observed in PM isolated from HERC2-overexpressing HCC cells (Fig. 7G and 7H). We then established PTP1B functional domain mutant plasmids based on a previous study [31] to investigate the specific domain that interacted with HERC2. Interestingly, only when PTP1B reserved the ER targeting domain did it interact with HERC2 (Fig. 7I). Overall, we concluded that HERC2 limits the interaction between PTP1B and JAK2 by inhibiting PTP1B translocation to ER-PM contact sites.

### *HERC2 promoted tumorigenesis and immune evasion of HCC *in vivo

**Fig. 8:**
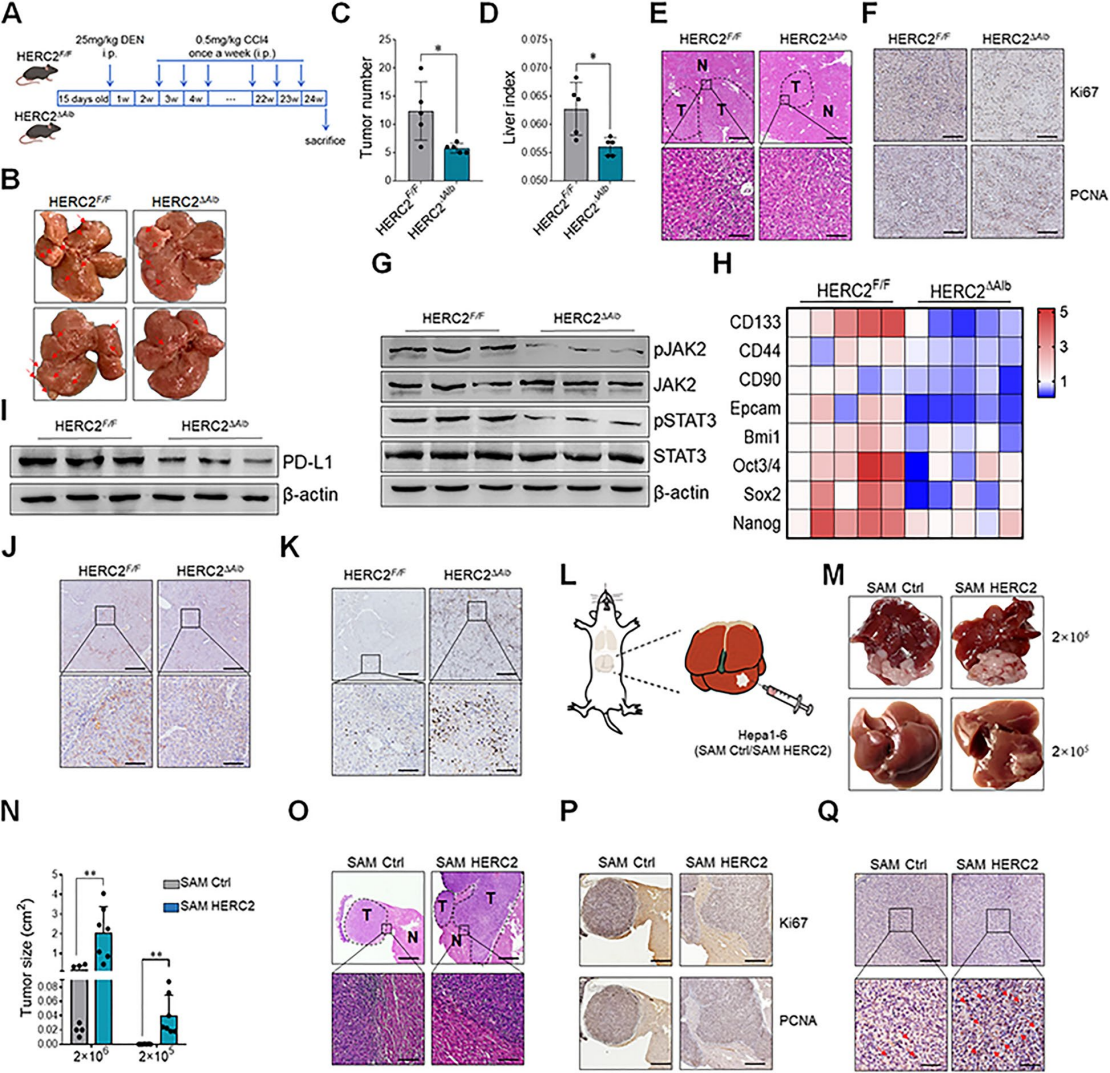
HERC2 promoted hepatic STAT3 activation and facilitated HCC tumorigenesis in vivo. **A** Diagram for mouse primary HCC induction. 15-day-old male mice were intraperitoneally injected with 25 μg/g DEN. Two weeks later, the mice were intraperitoneally injected with 0.5 μl/g CCl4 once a week for consecutive 22 weeks. **B** Tumor formation in liver tissues was observed, and the red arrow indicates typical tumor nodes. **C** The statistical diagram for tumor numbers is presented. **D** The statistical diagram for the liver index is presented. (**E**) H&E staining was performed to examine the pathological changes in liver tissues. Top scale bars = 250 μm, and bottom scale bars = 25 μm. **F** The expression of Ki67 and PCNA in liver tissues was determined by immunohistochemical assay. Scale bars = 100 μm. **G** Western blot analysis was performed to detect JAK2/STAT3 signaling activation. **H** RT-qPCR analysis was conducted to evaluate the levels of cancer stem cell-related genes. **I** Western blot analysis was performed to detect PD-L1 expression in liver tissues. **J** PD-L1 expression was evaluated by immunohistochemical assay. Top scale bars = 250 μm, and bottom scale bars = 50 μm. **K** The expression of CD8 was determined by immunohistochemical assay. Top scale bars = 250 μm, and bottom scale bars = 50 μm. **L-N** A total of 2 × 10^6^ or 2 × 10^5^ HERC2-overexpressing Hepa1-6 cells or control cells were orthotopically injected into mouse livers. **L** A diagram of mouse orthotopically implanted HCC is presented. **M** Tumor formation in liver tissues is presented. **N** A statistical diagram for liver tumor volume is presented. **O-Q** A total of 2 × 10^6^ HERC2-overexpressing Hepa1-6 cells or control cells were orthotopically injected into mouse livers. **O** H&E staining was performed to examine the pathological changes in liver tissues. Top scale bars = 250 μm, and bottom scale bars = 25 μm. **P** The expression of Ki67 and PCNA in liver tissues was determined by immunohistochemical assay. Scale bars = 250 μm. **Q** CD133 expression in liver tissues was determined by immunohistochemical assay. Top scale bars = 100 μm, and bottom scale bars = 25 μm. *p < 0.05, **p < 0.01. Data from one representative experiment of three independent experiments are presented

To further study the effect of HERC2 on HCC tumorigenesis in vivo, hepatocyte-specific HERC2 knockout mice (HERC2^∆Alb^) were established (Supplementary Fig. S8) and used to establish an inflammation-related HCC model (Fig. 8A). We observed decreased liver tumor amounts (Fig. 8B and C) and liver index (Fig. 8D) in HERC2^∆Alb^ mice compared to control mice. Attenuated tumorigenesis was displayed in HERC2^∆Alb^ mice compared to their counterparts, as determined by pathological analysis (Fig. 8E and F). Western blot analysis showed declined JAK2/STAT3 activation in liver tissues obtained from HERC2^∆Alb^ mice (Fig. 8G), and decreased expression of stemness-related genes was detected in livers from HERC2^∆Alb^ mice (Fig. 8H). We also tested PD-L1 expression in liver tissues and found restricted PD-L1 levels in HERC2^∆Alb^ mice compared to counterparts (Fig. 8I and J). Consistently, abundant CD8^+^ T cells were accumulated in liver tissues from HERC2^∆Alb^ mice compared to those from control mice (Fig. 8K). To further determine the role of HERC2 in HCC progression, HERC2-overexpressing Hepa1-6 cells and control cells were orthotopically injected into mouse liver to generate an orthotopic transplantation HCC model (Fig. 8L). Increased tumor size was observed in mice injected with diluted HERC2-overexpressing HCC cells compared to their counterparts (Fig. 8M and N). Aggravated tumorigenesis in HERC2-overexpressing cell-treated mice was also confirmed by pathological analysis (Fig. 8O and P). We further observed enhanced CD133 expression in liver tissues from HERC2-overexpressing cells injected into mice, as indicated by immunohistochemistry analysis (Fig. 8Q). Collectively, these data suggested that HERC2 enhanced hepatic STAT3 activation, presumably contributing to accelerated liver tumorigenesis in vivo.

## Discussion

**Fig. 9:**
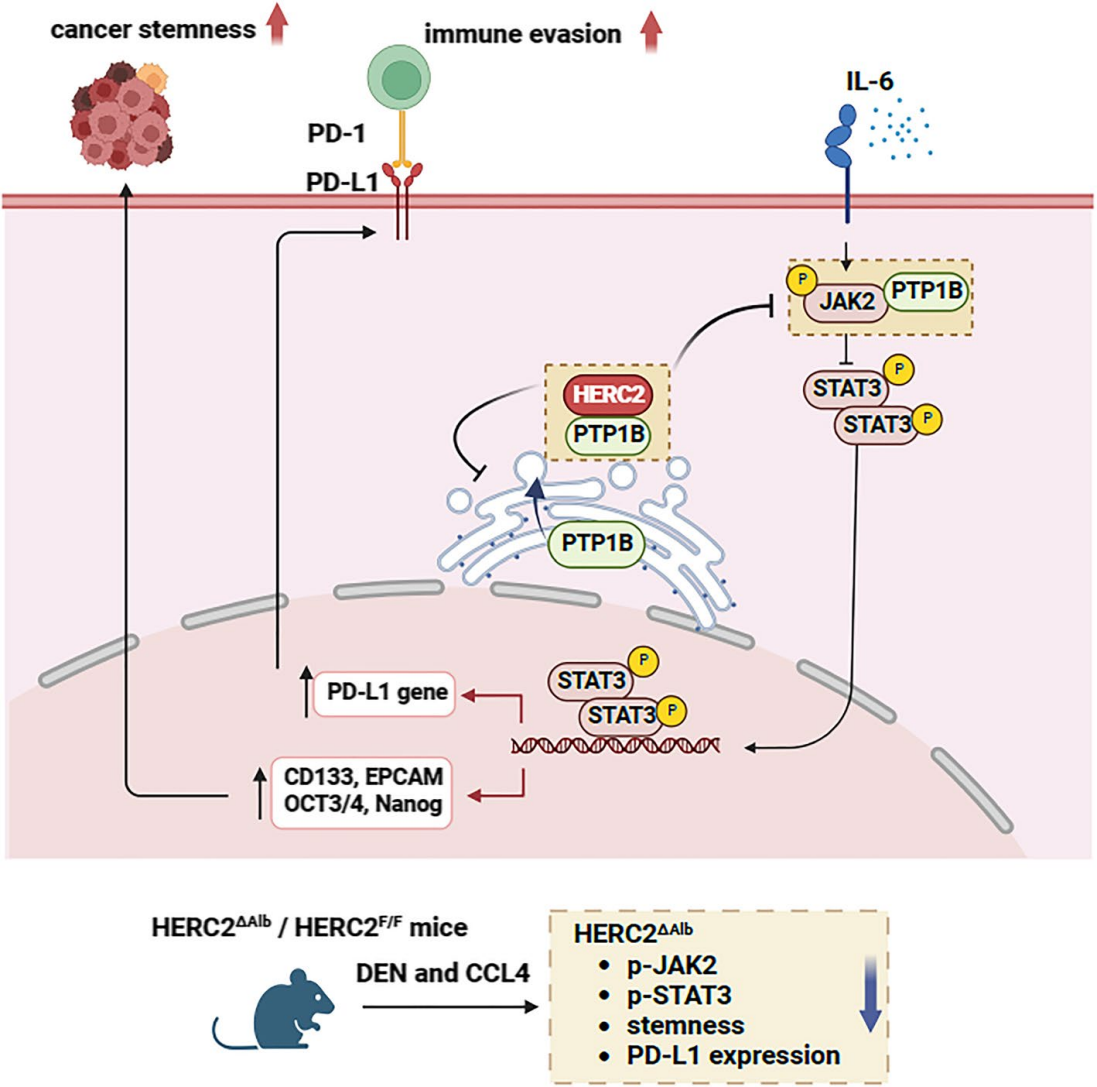
Schematic representation of the mechanism by which HERC2 promotes inflammation-related HCC progression. Inflammatory stimulation induces HERC2 expression in hepatocytes. HERC2 interacts with the C-terminal of PTP1B and limits PTP1B translocation in ER-plasma membrane junctions, thereby inhibiting PTP1B-mediated dephosphorylation of JAK2. The enhanced JAK2 phosphorylation activates STAT3 phosphorylation. Consequently, the overactivation of STAT3 is correlated with increased expression of stemness-related genes and PD-L1, further promoting cancer stemness and immune evasion in HCC

HCC commonly develops in a background of chronic inflammation. The prolonged expression of proinflammatory cytokines and chemokines promotes the malignant transformation of hepatocytes. In this study, we observed that inflammatory stimulation increased HERC2 expression in hepatocytes and that elevated HERC2 expression was associated with the progression and poor prognosis of HCC. Interestingly, HERC2 inhibited PD-L1 expression in HCC cells, which might be associated with the immune evasion of HCC. Moreover, we demonstrated that HERC2 promoted the stemness and immune evasion of HCC cells via JAK2/STAT3 signaling. Mechanistically, HERC2 interacted with PTP1B and inhibited PTP1B coupling with JAK2 at ER-PM junctions, thereby promoting STAT3 signaling activation (Fig. 9).

HERC2 belongs to the large HERC family of ubiquitin E3 ligases implicated in DNA repair regulation, neurodevelopment, and inflammation [16]. HERC2-mediated breast cancer gene 1 (BRCA1) degradation is involved in DNA double-strand breaks repair, thereby enhancing breast cancer cell proliferation [17]. Lomi et al*.* found that HERC2 could be used as a predictive factor in uveal melanoma with high pigmentation, which exhibited high metastatic potential [32]. Herein, we detected elevated HERC2 levels in HCC cells upon inflammatory stimulation, and that HERC2 overexpression promoted the proliferation and migration of HCC cells. The observation is correlated with the GSE21031 datasets that exhibited increased HERC2 expression in IL-6-stimulated primary mouse hepatocytes [33]. Our findings revealed a vital role of HERC2 in the process of inflammation-related HCC tumorigenesis. Notably, ChIP-seq analysis showed that STAT3 binds to the promoter of HERC2 based on the Cistrome DB database, indicating that HERC2 is a potential target gene of STAT3 that inflammatory cytokines can induce. This study showed that inflammatory stimulus-induced HERC2 expression in hepatocytes and that HERC2 significantly enhanced STAT3 phosphorylation. Therefore, we assumed that HERC2 might participate in the feed-forward STAT3-activation signaling loop that drives HCC tumorigenesis, which still needs further demonstration.

Expansion of CSCs is thought to be the initial stage of cancer development [9]. In our study, we found that HERC2 enhanced the stemness of HCC cells, including high expression of stemness-related markers, accelerated self-renewal ability, and increased resistance to sorafenib-induced apoptosis. Saez. et al. found that HERC2 is one of the most elevated E3 ubiquitin ligases in human embryonic stem cells (hESCs) compared to their differentiated counterparts [34]. Indeed, the highly abundant E3 enzymes interact with stem cell regulators in hESCs. Notch signaling has been demonstrated to be critical in the regulation of stem cells to differentiated cell fates [35, 36]. HERC2 interacts with leucine-rich repeat kinase 2 (LRRK2) to facilitate the recycling of the Notch ligand Delta-like 1, consequently accelerating neural stem cell differentiation [37]. McMillan et al. determined that HERC2 promotes ubiquitination of Notch ligands through its Mind bomb (Mib) domain [38]. Furthermore, exome sequencing data showed rare mutations in the HERC2 and Notch2 genes in individuals with central precocious puberty [39]. We postulated that the crosstalk between HERC2 and its interacting partners is essential for stem cell regulation.

T-cell exhaustion plays a vital role in the suppressive tumor microenvironment and tumor immune evasion. Emerging evidence supports the opinion that PD-L1 engagement on cancer cells with its receptor, PD-1, on effector T cells is the major mechanism contributing to the exhaustion of tumor-infiltrating CD8^+^ T cells and subsequent tumor immune evasion. Moreover, PD-1 expression was also observed in CD4^+^ T cells and CD56^+^ NK cells. A gene signature of cytotoxic CD4^+^ T cells in tumors predicts a clinical response in patients treated with anti-PD-L1 therapy [40]. Both PD-1 and PD-L1 blockade elicited a potent NK cell response as well [41].The US Food and Drug Administration (FDA) approved anti-PD-1 therapy has become a promising immunotherapy strategy for HCC. However, single-agent anti-PD-1 therapy did not reach a predetermined benefit in recent trials [42, 43]. Recent advances have transformed the focus on targeting immune checkpoints, such as PD-L1, to restore anti-tumor immune response [44]. The present study showed a significant correlation between HERC2 and PD-L1 expression in HCC. We observed that HERC2 increased the expression of PD-L1 in HCC cells, unveiling a critical role of HERC2 in the tumor immune microenvironment. Furthermore, our study implicated that HERC2 induced PD-L1 expression by activating STAT3, which is consistent with a previous research that showed that STAT3 increases PD-L1 expression via direct interaction with the PD-L1 promoter [45]. Additionally, we employed a coculture system in which activated PBMCs from healthy donors were cocultured with HCC cells in vitro. The result demonstrated the inhibitory effect of HERC2 on anti-tumor immunity, as indicated by impaired activation of CD8^+^ T cells, CD4^+^ T cells, and NK cells in the coculture system of PBMCs with HERC2-overexpressed HCC cells. Our study identified that HERC2 serves as an opposing force actively participating in the homeostatic control of PD-L1 in HCC, and interfering with the expression or function of HERC2 might help promote the anti-tumor efficiency of immune checkpoint-based therapy.

JAK2/STAT3 signaling is aberrantly hyperactivated in various cancers, and such hyperactivation is commonly associated with a poor clinical prognosis [46]. It should be noted that STAT3 activation affects both cancer stemness and immune evasion, which have emerged as important features of HCC initiation, development, and metastasis [47, 48]. Indeed, cancer stemness and immune evasion are closely associated and play crucial roles in tumor development [22]. Cancer stemness has been shown to be closely associated with the intrinsic immunosuppressive tumor microenvironment [48]. PD-L1 has functional roles in cancer stem-like cell phenotypes and chemoresistance besides immune evasion [22, 49]. We assumed that the regulatory effect of HERC2 on STAT3 signaling participates in the crosstalk between cancer stemness and immune evasion. Notably, STAT3 activation could be regulated by diverse protein tyrosine phosphatases that directly target JAKs or STAT3 [50]. We provided the first biochemical evidence displaying the interaction between HERC2 and PTP1B, which is able to dephosphorylate activated JAK2 and STAT3. Indeed, JAK2 is a substrate of PTP1B, and PTP1B deficiency causes the increased phosphorylation of JAK2 [25, 51]. It should be noted that PTP1B is anchored on the external face of the ER, and its activity is partly dependent on ER distribution and dynamics [52]. ER-PM junctions are contact sites between the ER membranes and the plasma membrane, where the two membranes are located in a close position and build a restricted cytoplasmic region [53]. The junction provides an ideal platform for lipid homeostasis, ion dynamics, and cell signaling [54]. Indeed, a number of proteins localize in crowded ER-PM junctions and act synergistically to maintain the local microenvironment. PTP1B is anchored to the surface of the ER membrane via its C-terminal fragment composed of 35 proline-rich residues. Anderie et al*.* demonstrated that PTP1B plays a catalytic function at ER-PM junctions by dephosphorylating its substrates located on the plasma membrane through the cytosolic catalytic domain [55]. JAK2, an essential physiological regulator of cytokine signaling in the plasma membrane, is one of the substrates of PTP1B [25]. Herein, the results showed that HERC2 significantly restricted the association of PTP1B with JAK2 in hepatocytes upon inflammatory stimulation. Most importantly, we provided evidence that HERC2 interacted with the ER-targeting domain of PTP1B and inhibited PTP1B translocation to ER-PM junctions. We assumed that HERC2 might function as a regulator supporting the communication of signaling proteins between the ER and the plasma membrane at ER-PM junctions. In this study, we observed HERC2 interacted with several ER-resident proteins based on our mass spectrum analysis. It is still worth investigating whether HERC2 affects the protein quality control of ER-resident protein and the potential role of HERC2 in ER homeostasis. The ubiquitin–proteasome system played an essential role in protein quality control, by which cells evolved dynamic and self-regulating quality control processes to adapt to new environmental conditions and prevent prolonged damage [56]. The E3 ligases have been classified into three families, the HECT-type, RING-type, and U-box-type E3 ligases [57]. HERC2 belongs to the HECT-type E3 ubiquitin family that mediated protein degradation [26, 27]. The RING-type ubiquitin ligase tripartite motif 18 (TRIM18) and U-box-type ubiquitin ligase precursor RNA processing-19β (PRP19β) have been reported to regulate PTP1B levels by ubiquitin-dependent proteasomal degradation under STAT3 activation [58, 59]. However, it seems that the HECT-type E3 ubiquitin ligases did not degrade PTP1B protein since HERC2 exhibited no influence on PTP1B protein level in hepatocytes upon inflammatory stimulation. A previous proteomic analysis determined potential HERC2 interaction networks of distinct cellular functions, consisting of regulating intracellular protein transport and trafficking [60]. Significantly, PTP1B can interact with diverse substrates, including cytosolic, nuclear, plasma membrane-bound, mitochondrial, and adherent junction proteins [61]. It might be a common phenomenon that HERC2 limits the connection of PTP1B with its substrates associated with the cytosolic face of the plasma membrane. Further research is warranted to determine whether PTP1B substrates are involved in HERC2-mediated inflammatory regulation of the STAT3 signaling pathway.

## Conclusions

In summary, we have demonstrated that HERC2 overexpression in hepatocytes enhances the development of HCC. The data showed that inflammatory stimulation significantly promotes HERC2 expression in hepatocytes. In turn, HERC2 modulates the dynamics of PTP1B and limits the function of PTP1B in regulating the STAT3 signaling pathway, thereby amplifying the STAT3-activation signaling feedback loop. Consequently, hyperactivated STAT3 signaling leads to stem-like genes and PD-L1 expression, eventually contributing to HCC development. In conclusion, our study identified HERC2 as a novel regulator for cancer stemness and immune evasion in inflammation-related HCC. HERC2 might be a potential therapeutic target for HCC immunotherapy, alone or in combination with anti-PD-L1/PD-1 antibodies.

## Supplementary Information

**Additional file 1: Supplementary Fig. S1**. IL-6-induced HERC2 expression is associated with HCC progression. **Supplementary Fig. S2.** HERC2 promoted the immune evasion of HCC cells. **Supplementary Fig. S3.** HERC2-positive tumor cells displayed higher STAT3-targeted gene expression. **Supplementary Fig. S4.** HERC2 promoted malignancy of HCC cells via STAT3 signaling. **Supplementary Fig. S5.** HERC2 enhanced stemness and immune evasion of HCC cells through STAT3 signaling. **Supplementary Fig. S6.** HERC2 promoted malignancy of HCC cells through PTP1B. **Supplementary Fig. S7.** HERC2 promoted stemness and immune evasion of HCC cells through PTP1B. **Supplementary Fig. S8.** Establishment of hepatocyte-specific HERC2 knockout mice.

**Additional file 2: Supplemental Video.** HERC2 limited PTP1B translocation in ER-PM junctions (related to Fig.6F). HEK293T cells expressing PTP1B-mCherry (Red) and Sec61β-GFP (Green). After transfection with HERC2 or mock plasmids, the cells were treated with 50 ng/ml IL-6. The cells were imaged every 40 seconds over a period of 10 minutes.

## Data Availability

All data needed to evaluate the conclusions in the paper are present in the paper and/or the supplementary material.

## References

[1] Yu LX, Ling Y, Wang HY. Role of nonresolving inflammation in hepatocellular carcinoma development and progression. NPJ Precis Oncol. 2018;2(1):6. 29872724 10.1038/s41698-018-0048-z PMC5871907

[2] Refolo MG, Messa C, Guerra V, Carr BI, D’Alessandro R. Inflammatory Mechanisms of HCC Development. Cancers (Basel). 2020;12(3):641. 32164265 10.3390/cancers12030641 PMC7139884

[3] Koyama Y, Brenner DA. Liver inflammation and fibrosis. J Clin Invest. 2017;127(1):55–64. 28045404 10.1172/JCI88881 PMC5199698

[4] Bergmann J, Muller M, Baumann N, Reichert M, Heneweer C, Bolik J, et al. IL-6 trans-signaling is essential for the development of hepatocellular carcinoma in Mice. Hepatology. 2017;65(1):89–103. 27770462 10.1002/hep.28874

[5] Budhu A, Wang XW. The role of cytokines in hepatocellular carcinoma. J Leukoc Biol. 2006;80(6):1197–213. 16946019 10.1189/jlb.0506297

[6] Porta C, Amici M, Quaglini S, Paglino C, Tagliani F, Boncimino A, et al. Circulating interleukin-6 as a tumor marker for hepatocellular carcinoma. Ann Oncol. 2008;19(2):353–8. 17962206 10.1093/annonc/mdm448

[7] Park EJ, Lee JH, Yu GY, He G, Ali SR, Holzer RG, et al. Dietary and genetic obesity promote liver inflammation and tumorigenesis by enhancing IL-6 and TNF expression. Cell. 2010;140(2):197–208. 20141834 10.1016/j.cell.2009.12.052 PMC2836922

[8] Zhao B, Wang Y, Tan X, Ke K, Zheng X, Wang F, et al. Inflammatory micro-environment contributes to Stemness properties and metastatic potential of HCC via the NF-kappaB/miR-497/SALL4 Axis. Mol Ther Oncolytics. 2019;15:79–90. 31650028 10.1016/j.omto.2019.08.009 PMC6804787

[9] Lee TK, Guan XY, Ma S. Cancer stem cells in hepatocellular carcinoma - from origin to clinical implications. Nat Rev Gastroenterol Hepatol. 2022;19(1):26–44. 34504325 10.1038/s41575-021-00508-3

[10] Nguyen PHD, Wasser M, Tan CT, Lim CJ, Lai HLH, Seow JJW, et al. Trajectory of immune evasion and cancer progression in hepatocellular carcinoma. Nature Commun. 2022;13(1):1441. 35301339 10.1038/s41467-022-29122-w PMC8931110

[11] Zhao HK, Wu L, Yan GF, Chen Y, Zhou MY, Wu YZ, et al. Inflammation and tumor progression: signaling pathways and targeted intervention. Signal Transduct Tar. 2021;6(1):263. 10.1038/s41392-021-00658-5 PMC8273155 34248142

[12] Lim SO, Li CW, Xia W, Cha JH, Chan LC, Wu Y, et al. Deubiquitination and stabilization of PD-L1 by CSN5. Cancer Cell. 2016;30(6):925–39. 27866850 10.1016/j.ccell.2016.10.010 PMC5171205

[13] Zhong F, Cheng X, Sun S, Zhou J. Transcriptional activation of PD-L1 by Sox2 contributes to the proliferation of hepatocellular carcinoma cells. Oncol Rep. 2017;37(5):3061–7. 28339084 10.3892/or.2017.5523

[14] Uhlig J, Stein S, Kim HS. PD-1 targeted immunotherapy for advanced hepatocellular carcinoma: current utilization and outcomes in the USA. Future Oncol. 2022;18(14):1691–703. 35172633 10.2217/fon-2021-1487

[15] Cheng AL, Hsu C, Chan SL, Choo SP, Kudo M. Challenges of combination therapy with immune checkpoint inhibitors for hepatocellular carcinoma. J Hepatol. 2020;72(2):307–19. 31954494 10.1016/j.jhep.2019.09.025

[16] Garcia-Cano J, Martinez-Martinez A, Sala-Gaston J, Pedrazza L, Rosa JL. HERCing: structural and functional relevance of the large HERC ubiquitin ligases. Front Physiol. 2019;10:1014. 31447701 10.3389/fphys.2019.01014 PMC6692442

[17] Wu W, Sato K, Koike A, Nishikawa H, Koizumi H, Venkitaraman AR, et al. HERC2 is an E3 ligase that targets BRCA1 for degradation. Cancer Res. 2010;70(15):6384–92. 20631078 10.1158/0008-5472.CAN-10-1304

[18] Lee TH, Park JM, Leem SH, Kang TH. Coordinated regulation of XPA stability by ATR and HERC2 during nucleotide excision repair. Oncogene. 2014;33(1):19–25. 23178497 10.1038/onc.2012.539

[19] Ramakrishna G, Rastogi A, Trehanpati N, Sen B, Khosla R, Sarin SK. From cirrhosis to hepatocellular carcinoma: new molecular insights on inflammation and cellular senescence. Liver Cancer. 2013;2(3–4):367–83. 24400224 10.1159/000343852 PMC3881319

[20] Won C, Kim BH, Yi EH, Choi KJ, Kim EK, Jeong JM, et al. Signal transducer and activator of transcription 3-mediated CD133 up-regulation contributes to promotion of hepatocellular carcinoma. Hepatology. 2015;62(4):1160–73. 26154152 10.1002/hep.27968 PMC5049669

[21] Sanmamed MF, Chen L. A paradigm shift in cancer immunotherapy: from enhancement to normalization. Cell. 2018;175(2):313–26. 30290139 10.1016/j.cell.2018.09.035 PMC6538253

[22] Hsu JM, Xia W, Hsu YH, Chan LC, Yu WH, Cha JH, et al. STT3-dependent PD-L1 accumulation on cancer stem cells promotes immune evasion. Nat Commun. 2018;9(1):1908. 29765039 10.1038/s41467-018-04313-6 PMC5954021

[23] Bourdeau A, Dube N, Tremblay ML. Cytoplasmic protein tyrosine phosphatases, regulation and function: the roles of PTP1B and TC-PTP. Curr Opin Cell Biol. 2005;17(2):203–9. 15780598 10.1016/j.ceb.2005.02.001

[24] Cook WS, Unger RH. Protein tyrosine phosphatase 1B: a potential leptin resistance factor of obesity. Dev Cell. 2002;2(4):385–7. 11970889 10.1016/s1534-5807(02)00158-2

[25] Myers MP, Andersen JN, Cheng A, Tremblay ML, Horvath CM, Parisien JP, et al. TYK2 and JAK2 are substrates of protein-tyrosine phosphatase 1B. J Biol Chem. 2001;276(51):47771–4. 11694501 10.1074/jbc.C100583200

[26] Perez-Villegas EM, Ruiz R, Bachiller S, Ventura F, Armengol JA, Rosa JL. The HERC proteins and the nervous system. Semin Cell Dev Biol. 2022;132:5–15. 10.1016/j.semcdb.2021.11.017 34848147

[27] Sanchez-Tena S, Cubillos-Rojas M, Schneider T, Rosa JL. Functional and pathological relevance of HERC family proteins: a decade later. Cell Mol Life Sci. 2016;73(10):1955–68. 26801221 10.1007/s00018-016-2139-8 PMC11108380

[28] Garcia-Cano J, Sanchez-Tena S, Sala-Gaston J, Figueras A, Vinals F, Bartrons R, et al. Regulation of the MDM2-p53 pathway by the ubiquitin ligase HERC2. Mol Oncol. 2020;14(1):69–86. 31665549 10.1002/1878-0261.12592 PMC6944118

[29] Lees JA, Messa M, Sun EW, Wheeler H, Torta F, Wenk MR, et al. Lipid transport by TMEM24 at ER-plasma membrane contacts regulates pulsatile insulin secretion. Science. 2017;355(6326):eaah6171. 28209843 10.1126/science.aah6171 PMC5414417

[30] Giordano F, Saheki Y, Idevall-Hagren O, Colombo SF, Pirruccello M, Milosevic I, et al. PI(4,5)P(2)-dependent and Ca(2+)-regulated ER-PM interactions mediated by the extended synaptotagmins. Cell. 2013;153(7):1494–509. 23791178 10.1016/j.cell.2013.05.026 PMC3716012

[31] Lee D, Kraus A, Prins D, Groenendyk J, Aubry I, Liu WX, et al. UBC9-dependent association between calnexin and protein tyrosine phosphatase 1B (PTP1B) at the endoplasmic reticulum. J Biol Chem. 2015;290(9):5725–38. 25586181 10.1074/jbc.M114.635474 PMC4342483

[32] Kashyap S, Singh MK, Jha J, Singh L, Pushker N, Sen S, et al. Prognostic impact of HERC2 protein and pink-eyed dilution protein in uveal melanoma. Hum Cell. 2020;33(4):1264–72. 32686068 10.1007/s13577-020-00397-9

[33] Kowarsch A, Blochl F, Bohl S, Saile M, Gretz N, Klingmuller U, et al. Knowledge-based matrix factorization temporally resolves the cellular responses to IL-6 stimulation. BMC Bioinformatics. 2010;11:585. 21118515 10.1186/1471-2105-11-585 PMC3009690

[34] Saez I, Koyuncu S, Gutierrez-Garcia R, Dieterich C, Vilchez D. Insights into the ubiquitin-proteasome system of human embryonic stem cells. Sci Rep. 2018;8(1):4092. 29511261 10.1038/s41598-018-22384-9 PMC5840266

[35] Takebe N, Harris PJ, Warren RQ, Ivy SP. Targeting cancer stem cells by inhibiting Wnt, Notch, and Hedgehog pathways. Nat Rev Clin Oncol. 2011;8(2):97–106. 21151206 10.1038/nrclinonc.2010.196

[36] Majumder S, Crabtree JS, Golde TE, Minter LM, Osborne BA, Miele L. Targeting Notch in oncology: the path forward. Nat Rev Drug Discov. 2021;20(2):125–44. 33293690 10.1038/s41573-020-00091-3

[37] Imai Y, Kobayashi Y, Inoshita T, Meng H, Arano T, Uemura K, et al. The Parkinson’s disease-associated protein kinase LRRK2 modulates notch signaling through the endosomal pathway. PLoS Genet. 2015;11(9):e1005503. 26355680 10.1371/journal.pgen.1005503 PMC4565672

[38] McMillan BJ, Schnute B, Ohlenhard N, Zimmerman B, Miles L, Beglova N, et al. A tail of two sites: a bipartite mechanism for recognition of notch ligands by mind bomb E3 ligases. Mol Cell. 2015;57(5):912–24. 25747658 10.1016/j.molcel.2015.01.019 PMC4355479

[39] Lee HS, Jeong HR, Rho JG, Kum CD, Kim KH, Kim DW, et al. Identification of rare missense mutations in NOTCH2 and HERC2 associated with familial central precocious puberty via whole-exome sequencing. Gynecol Endocrinol. 2020;36(8):682–6. 32400230 10.1080/09513590.2020.1760241

[40] Oh DY, Kwek SS, Raju SS, Li T, McCarthy E, Chow E, et al. Intratumoral CD4(+) T cells mediate anti-tumor cytotoxicity in human bladder cancer. Cell. 2020;181(7):1612-25 e13. 32497499 10.1016/j.cell.2020.05.017 PMC7321885

[41] Hsu J, Hodgins JJ, Marathe M, Nicolai CJ, Bourgeois-Daigneault MC, Trevino TN, et al. Contribution of NK cells to immunotherapy mediated by PD-1/PD-L1 blockade. J Clin Invest. 2018;128(10):4654–68. 30198904 10.1172/JCI99317 PMC6159991

[42] Finn RS, Ryoo BY, Merle P, Kudo M, Bouattour M, Lim HY, et al. Pembrolizumab as second-line therapy in patients with advanced hepatocellular carcinoma in KEYNOTE-240: a randomized, double-blind. Phase III Trial J Clin Oncol. 2020;38(3):193–202. 31790344 10.1200/JCO.19.01307

[43] El-Khoueiry AB, Sangro B, Yau T, Crocenzi TS, Kudo M, Hsu C, et al. Nivolumab in patients with advanced hepatocellular carcinoma (CheckMate 040): an open-label, non-comparative, phase 1/2 dose escalation and expansion trial. Lancet. 2017;389(10088):2492–502. 28434648 10.1016/S0140-6736(17)31046-2 PMC7539326

[44] Topalian SL, Drake CG, Pardoll DM. Immune checkpoint blockade: a common denominator approach to cancer therapy. Cancer Cell. 2015;27(4):450–61. 25858804 10.1016/j.ccell.2015.03.001 PMC4400238

[45] Luo F, Luo M, Rong QX, Zhang H, Chen Z, Wang F, et al. Niclosamide, an antihelmintic drug, enhances efficacy of PD-1/PD-L1 immune checkpoint blockade in non-small cell lung cancer. J Immunother Cancer. 2019;7(1):245. 31511071 10.1186/s40425-019-0733-7 PMC6739982

[46] Johnson DE, O’Keefe RA, Grandis JR. Targeting the IL-6/JAK/STAT3 signalling axis in cancer. Nat Rev Clin Oncol. 2018;15(4):234–48. 29405201 10.1038/nrclinonc.2018.8 PMC5858971

[47] Zou S, Tong Q, Liu B, Huang W, Tian Y, Fu X. Targeting STAT3 in Cancer Immunotherapy. Mol Cancer. 2020;19(1):145. 32972405 10.1186/s12943-020-01258-7 PMC7513516

[48] Jia L, Wang Y, Wang CY. circFAT1 Promotes Cancer Stemness and Immune Evasion by Promoting STAT3 Activation. Adv Sci (Weinh). 2021;8(13):2003376. 34258151 10.1002/advs.202003376 PMC8261519

[49] Wei F, Zhang T, Deng SC, Wei JC, Yang P, Wang Q, et al. PD-L1 promotes colorectal cancer stem cell expansion by activating HMGA1-dependent signaling pathways. Cancer Lett. 2019;450:1–13. 30776481 10.1016/j.canlet.2019.02.022

[50] Huynh J, Chand A, Gough D, Ernst M. Therapeutically exploiting STAT3 activity in cancer - using tissue repair as a road map. Nat Rev Cancer. 2019;19(2):82–96. 30578415 10.1038/s41568-018-0090-8

[51] Tsunekawa T, Banno R, Mizoguchi A, Sugiyama M, Tominaga T, Onoue T, et al. Deficiency of PTP1B attenuates hypothalamic inflammation via activation of the JAK2-STAT3 pathway in Microglia. EBioMedicine. 2017;16:172–83. 28094236 10.1016/j.ebiom.2017.01.007 PMC5474442

[52] Hernandez MV, Sala MG, Balsamo J, Lilien J, Arregui CO. ER-bound PTP1B is targeted to newly forming cell-matrix adhesions. J Cell Sci. 2006;119(Pt 7):1233–43. 16522684 10.1242/jcs.02846

[53] Okeke E, Dingsdale H, Parker T, Voronina S, Tepikin AV. Endoplasmic reticulum-plasma membrane junctions: structure, function and dynamics. J Physiol. 2016;594(11):2837–47. 26939537 10.1113/JP271142 PMC4887688

[54] Carrasco S, Meyer T. STIM proteins and the endoplasmic reticulum-plasma membrane junctions. Annu Rev Biochem. 2011;80:973–1000. 21548779 10.1146/annurev-biochem-061609-165311 PMC3897197

[55] Anderie I, Schulz I, Schmid A. Direct interaction between ER membrane-bound PTP1B and its plasma membrane-anchored targets. Cell Signal. 2007;19(3):582–92. 17092689 10.1016/j.cellsig.2006.08.007

[56] Pohl C, Dikic I. Cellular quality control by the ubiquitin-proteasome system and autophagy. Science. 2019;366(6467):818–22. 31727826 10.1126/science.aax3769

[57] Ryu MY, Cho SK, Hong Y, Kim J, Kim JH, Kim GM, et al. Classification of barley U-box E3 ligases and their expression patterns in response to drought and pathogen stresses. BMC Genomics. 2019;20(1):326. 31035917 10.1186/s12864-019-5696-z PMC6489225

[58] Chen Q, Gao C, Wang M, Fei X, Zhao N. TRIM18-regulated STAT3 signaling pathway via PTP1B promotes renal epithelial-mesenchymal transition, inflammation, and fibrosis in diabetic kidney disease. Front Physiol. 2021;12:709506. 34434118 10.3389/fphys.2021.709506 PMC8381599

[59] Yamada T, Urano-Tashiro Y, Hashi Y, Sakumoto M, Akiyama H, Tashiro F. The U-box-type ubiquitin ligase PRP19beta regulates astrocyte differentiation via ubiquitination of PTP1B. Brain Res. 2013;1524:12–25. 23769735 10.1016/j.brainres.2013.06.007

[60] Galligan JT, Martinez-Noel G, Arndt V, Hayes S, Chittenden TW, Harper JW, et al. Proteomic analysis and identification of cellular interactors of the giant ubiquitin ligase HERC2. J Proteome Res. 2015;14(2):953–66. 25476789 10.1021/pr501005v PMC4324439

[61] Mertins P, Eberl HC, Renkawitz J, Olsen JV, Tremblay ML, Mann M, et al. Investigation of protein-tyrosine phosphatase 1B function by quantitative proteomics. Mol Cell Proteomics. 2008;7(9):1763–77. 18515860 10.1074/mcp.M800196-MCP200 PMC2556021

