# Correction: HERC2 promotes inflammation-driven cancer stemness and immune evasion in hepatocellular carcinoma by activating STAT3 pathway

**DOI:** 10.1186/s13046-024-03223-4

**Published:** 2024-11-13

**Authors:** Yunzhi Liu, Qishan Xu, Fan Deng, Zhuojun Zheng, Jialiang Luo, Ping Wang, Jia Zhou, Xiao Lu, Liyun Zhang, Zhengliang Chen, Qifan Zhang, Qingyun Chen, Daming Zuo

**Affiliations:** 1 Department of Medical Laboratory, School of Laboratory Medicine and Biotechnology, Southern Medical University, Guangzhou, Guangdong 510515 China; 2 Guangdong Province Key Laboratory of Proteomics, Department of Immunology, School of Basic Medical Sciences, Southern Medical University, Guangzhou, Guangdong 510515 China; 3 Clinical Oncology Center, Shenzhen Key Laboratory for Cancer Metastasis and Personalized Therapy, The University of Hong Kong-Shenzhen Hospital, Shenzhen, Guangdong 518053 China; 4 Shenzhen Institute of Advanced Technology, Chinese Academy of Sciences, Shenzhen, 518055 China; 5 Department of Hepatobiliary Surgery, Nanfang Hospital, Southern Medical University, Guangzhou, Guangdong 510515 China; 6 Medical Research Institute, Guangdong Provincial People’s Hospital, Guangdong Academy of Medical Sciences, Guangzhou, Guangdong 510080 China; 7 Guangdong Province Key Laboratory of Immune Regulation and Immunotherapy, School of Laboratory Medicine and Biotechnology, Southern Medical University, Guangzhou, Guangdong 510515 China; 8 Microbiome Medicine Center, Zhujiang Hospital, Southern Medical University, Guangzhou, Guangdong 510282 China

**Correction: J Exp Clin Cancer Res 42, 38 (2023)**

10.1186/s13046-023-02609-0

Following the publication of the original article [1], the author identified an error in Fig. 5. The western blot of Fig. 5D was inadvertently duplicated from the left panel. This occurred when the authors mistakenly overwrote the right panel data with the left panel data while organizing the raw files.

The correct figure is presented below:

**Incorrect** **Fig.** 5.

**Fig. 5:**
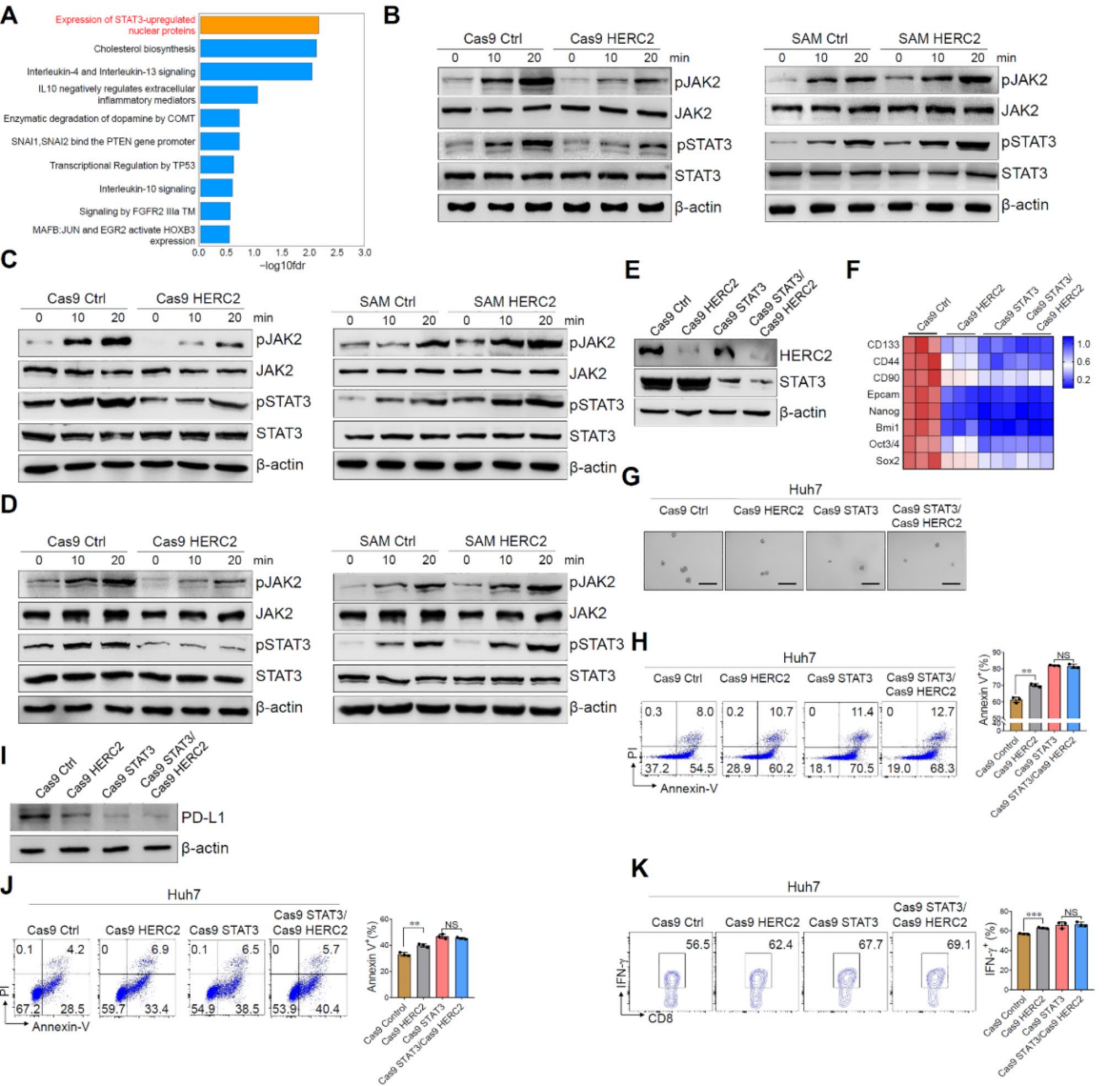
HERC2 promoted the stemness and immune evasion of HCC cells through JAK2/STAT3 signaling. **A** HERC2 knockout Huh7 cells were treated with 50 ng/ml IL-6 for 24 h and then subjected to RNA-seq analysis. Reactome pathway analysis displayed the most enriched pathways. **B**-**D** HERC2-defcient Huh7 cells and HERC2-overexpressing HCC-97 h cells were treated with 50 ng/ml IL-6 (**B**), 50 ng/ml IL-11 (**C**), or 20 ng/ml EGF (**D**), respectively. The phosphorylation of JAK2 and STAT3 was determined by western blot analysis. **E** HERC2 and STAT3 double-defcient Huh7 cell lines were established. **F** The cells were treated with 50 ng/ml IL-6 for 24 h. The RT-qPCR assay was used to detect the mRNA expression of cancer stem cell-related genes. **G** The cells were cultured in a conditioned medium with 100×N2, 50×B27, 20 ng/ml EGF, 10 nmol FGF, 5 µg/ml insulin, and 0.4% BSA for 7 days, scale bars = 100 μm. **H** The cells were treated with 20 µM sorafenib for 24 h. A fow cytometry assay was used to determine the percentage of apoptotic cells. **I** Cells were treated with 50 ng/ml IL-6 for 24 h and then subjected to western blot assay for PD-L1 detection. **J** and **K** Activated PBMCs were cocultured with HCC cells at the ratio of 4:1 for 24 h. Apoptosis of HCC cells was detected by fow cytometry assay (**J**). **K** IFN-γ levels of CD8 + T cells were determined by fow cytometry analysis. NS: not signifcant, **p < 0.01, ***p < 0.001. Data from one representative experiment of three independent experiments are presented

**Correct** Fig. 5.

**Fig. 5:**
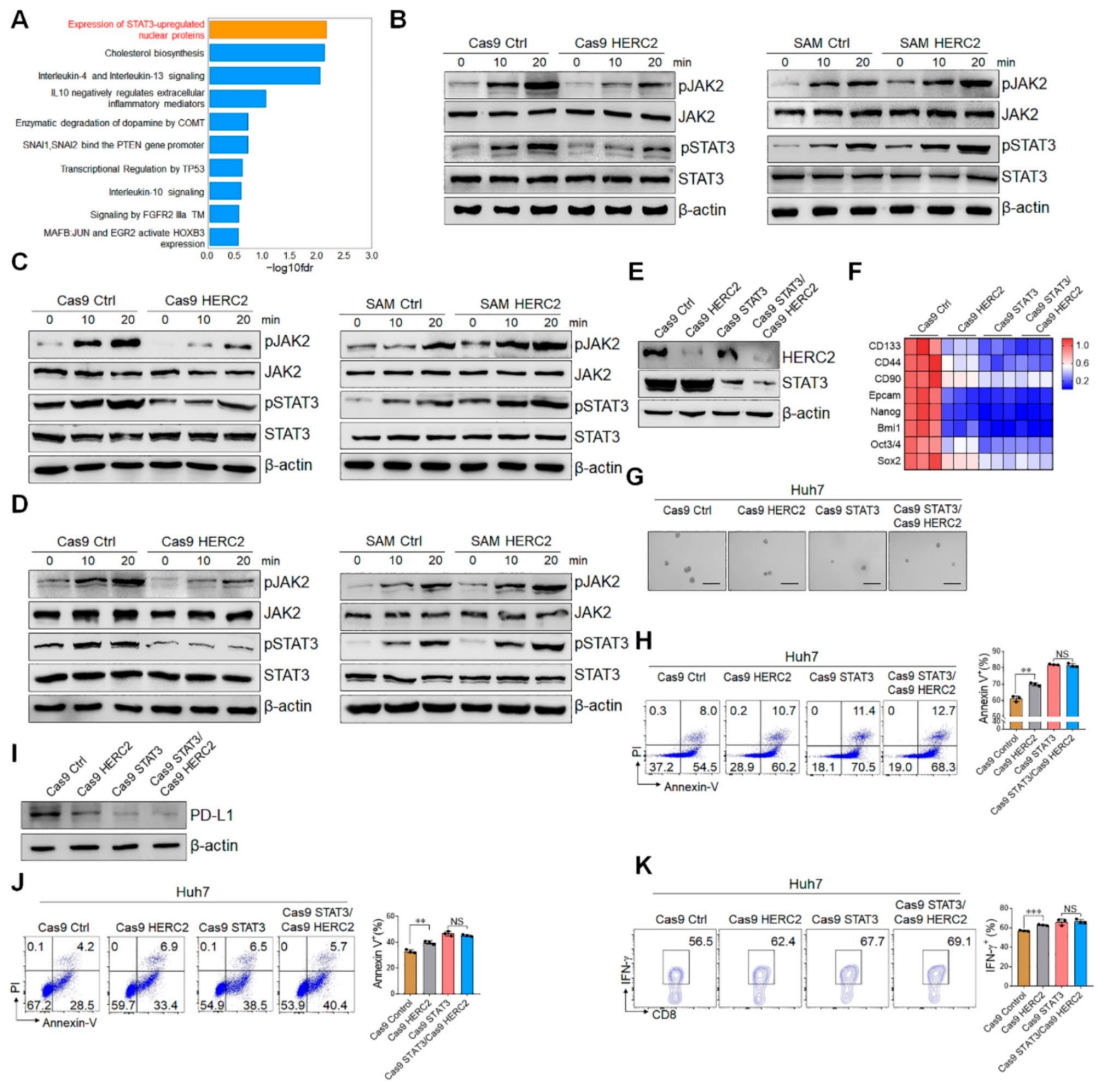
HERC2 promoted the stemness and immune evasion of HCC cells through JAK2/STAT3 signaling. **A** HERC2 knockout Huh7 cells were treated with 50 ng/ml IL-6 for 24 h and then subjected to RNA-seq analysis. Reactome pathway analysis displayed the most enriched pathways. **B**-**D** HERC2-defcient Huh7 cells and HERC2-overexpressing HCC-97 h cells were treated with 50 ng/ml IL-6 (**B**), 50 ng/ml IL-11 (**C**), or 20 ng/ml EGF (**D**), respectively. The phosphorylation of JAK2 and STAT3 was determined by western blot analysis. **E** HERC2 and STAT3 double-defcient Huh7 cell lines were established. **F** The cells were treated with 50 ng/ml IL-6 for 24 h. The RT-qPCR assay was used to detect the mRNA expression of cancer stem cell-related genes. **G** The cells were cultured in a conditioned medium with 100×N2, 50×B27, 20 ng/ml EGF, 10 nmol FGF, 5 µg/ml insulin, and 0.4% BSA for 7 days, scale bars = 100 μm. **H** The cells were treated with 20 µM sorafenib for 24 h. A fow cytometry assay was used to determine the percentage of apoptotic cells. **I** Cells were treated with 50 ng/ml IL-6 for 24 h and then subjected to western blot assay for PD-L1 detection. **J** and **K** Activated PBMCs were cocultured with HCC cells at the ratio of 4:1 for 24 h. Apoptosis of HCC cells was detected by fow cytometry assay (**J**). **K** IFN-γ levels of CD8 + T cells were determined by fow cytometry analysis. NS: not signifcant, **p < 0.01, ***p < 0.001. Data from one representative experiment of three independent experiments are presented

The correction does not compromise the validity of the conclusions and the overall content of the article. The original article [1] has been updated.
