# Supplementary material for: HERC2 promotes inflammation-driven cancer stemness and immune evasion in hepatocellular carcinoma by activating STAT3 pathway
Source: J Exp Clin Cancer Res. 2023 Feb 1;42:38. doi: 10.1186/s13046-023-02609-0 (PMC9890722; doi:10.1186/s13046-023-02609-0)
Supplement: Supplementary file 1 — Additional file 1: Supplementary Fig. S1. IL-6-induced HERC2 expression is associated with HCC progression. Supplementary Fig. S2. HERC2 promoted the immune evasion of HCC cells. Supplementary Fig. S3. HERC2-positive tumor cells displayed higher STAT3-targeted gene expression. Supplementary Fig. S4. HERC2 promoted malignancy of HCC cells via STAT3 signaling. Supplementary Fig. S5. HERC2 enhanced stemness and immune evasion of HCC cells through STAT3 signaling. Supplementary Fig. S6. HERC2 promoted malignancy of HCC cells through PTP1B. Supplementary Fig. S7. HERC2 promoted stemness and immune evasion of HCC cells through PTP1B. Supplementary Fig. S8. Establishment of hepatocyte-specific HERC2 knockout mice. [file 13046_2023_2609_MOESM1_ESM.docx]

**Supplementary Figure 1**

**
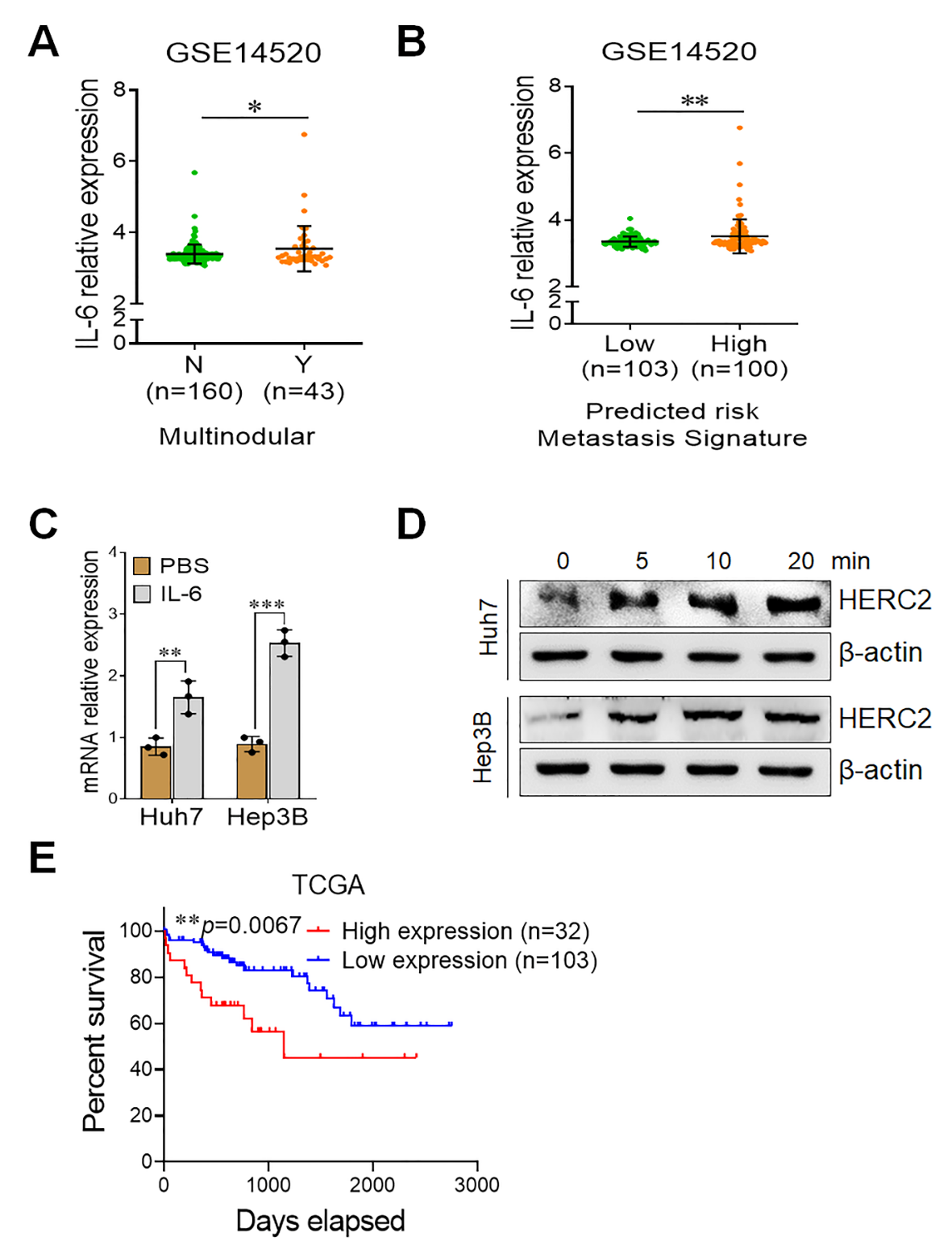
**

**Supplementary Fig. S1** **IL-6-induced HERC2 expression is associated with HCC progression.** (A) Expression levels of IL-6 in HCC patients with (n=43) or without (n=160) multinodular according to GSE14520 datasets. (B) Expression levels of IL-6 in HCC patients with high (n=100) or low (n=103) predicted risk metastasis signature according to GSE14520 datasets. (C) Hep3B and Huh7 HCC cell lines were stimulated with 50 ng/ml IL-6 for 20 minutes, and the mRNA levels of HERC2 were detected by RT-qPCR analysis. (D) Huh7 and Hep3B HCC cell lines were stimulated with 50 ng/ml IL-6 for the indicated time points, and the protein levels of HERC2 were evaluated by western blotting analysis. (E) Cox regression analysis of HCC patients with high HERC2 expression (n=32) and low HERC2 expression (n=103) based on TCGA datasets. **p*<0.05, **p<0.01, ***p<0.001.

**Supplementary Figure 2**


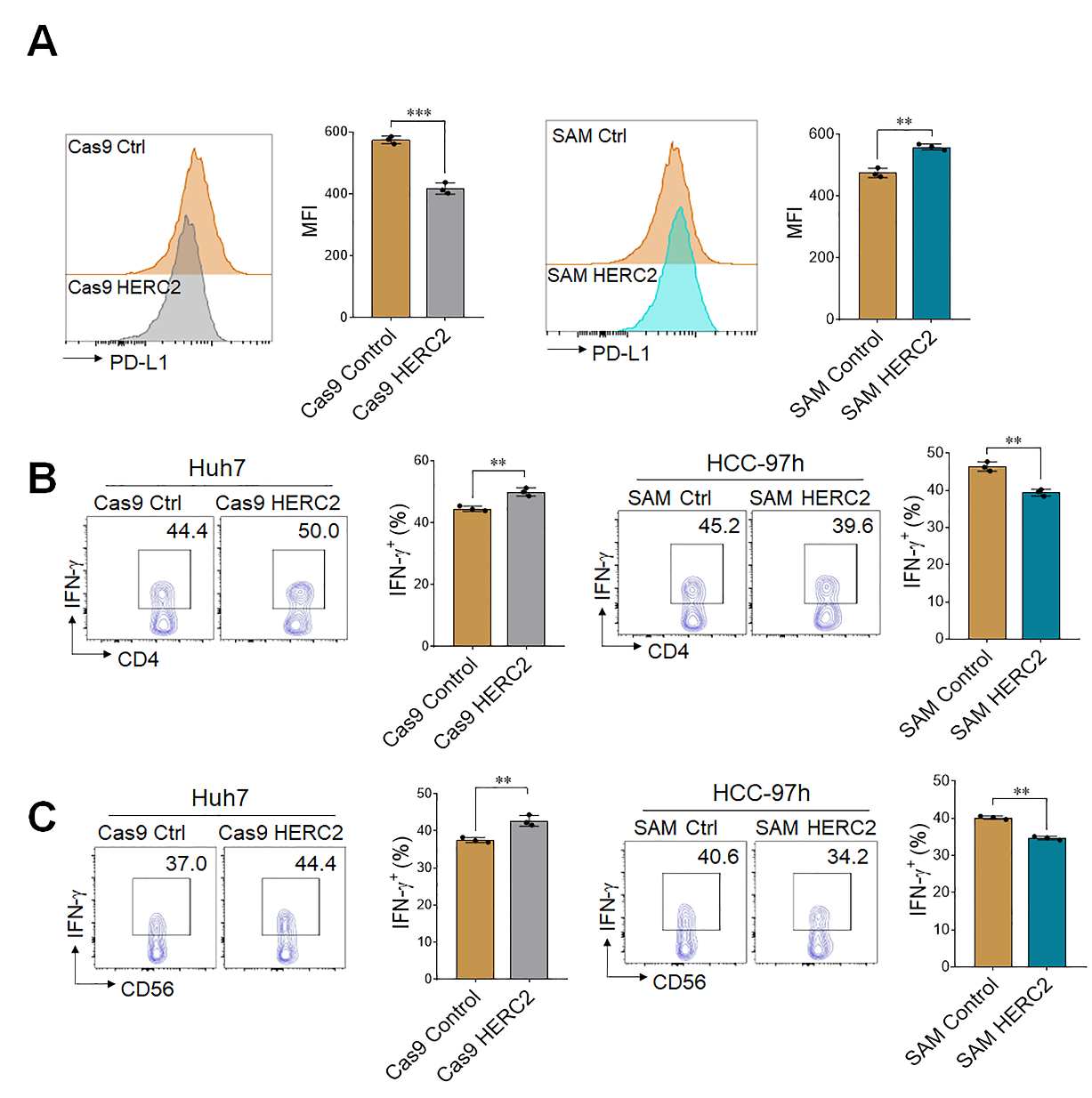


**Supplementary Fig. S2** **HERC2 promoted the immune evasion of HCC cells.** (A) HERC2-deficient Huh7 cells and HERC2-overexpressing HCC-97h cells were treated with 50 ng/ml IL-6 for 24 hours. Flow cytometry analysis was used to determine PD-L1 expression on the cell surface. (B-C) Activated PBMCs were cocultured with Huh7 cells or HERC2-overexpressing HCC-97h cells at the ratio of 4:1 for 24 hours. (B) IFN-γ levels of CD4^+^ T cells were determined by flow cytometry analysis. (C) IFN-γ levels of CD56^+^ NK cells were detected by flow cytometry assay. **p<0.01, ***p<0.001. Data from one representative experiment of three independent experiments are presented.

**Supplementary Figure 3**


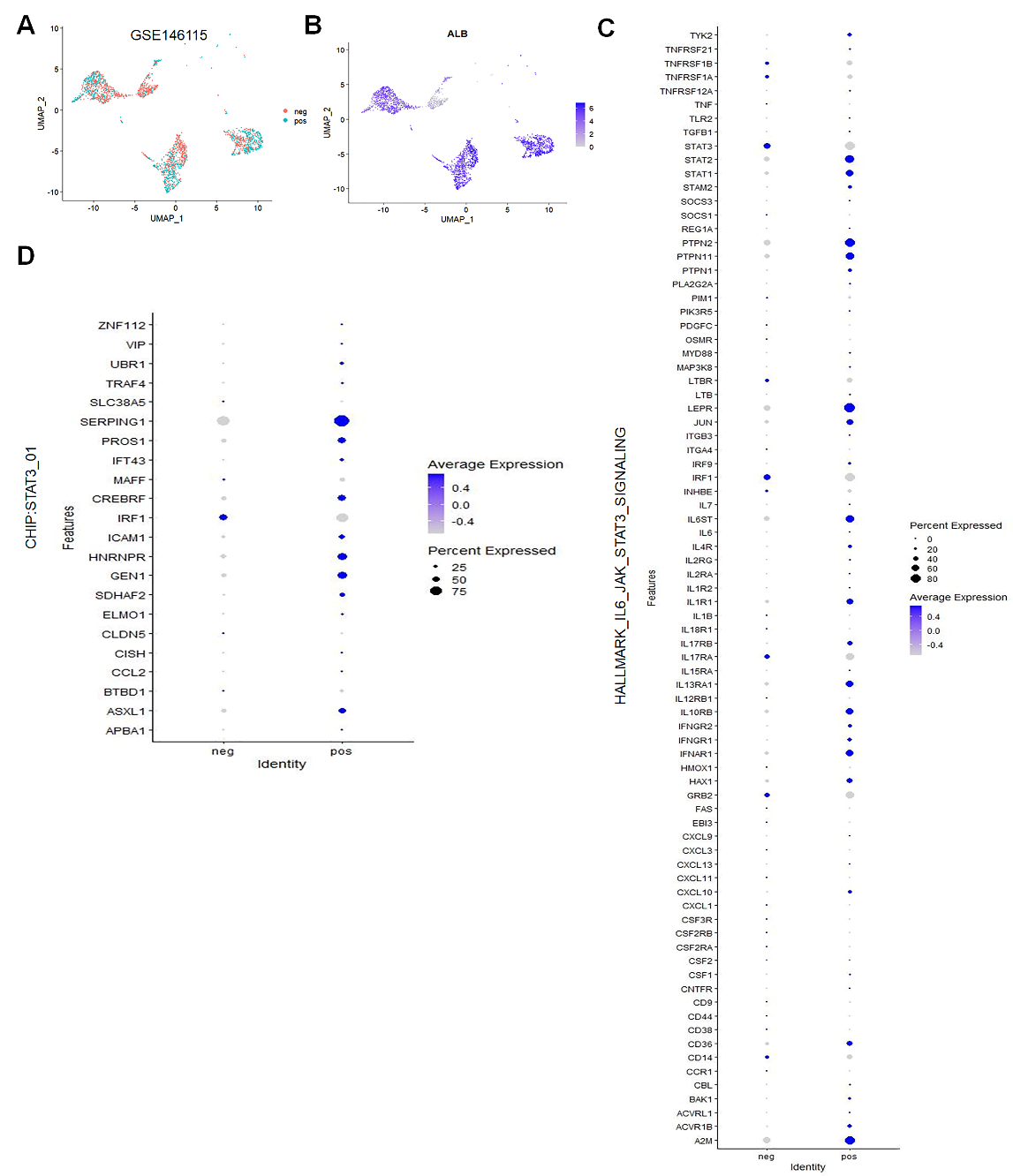


**Supplementary Fig. S3** **HERC2-positive tumor cells displayed higher STAT3-targeted gene expression.** HCC single cell RNA-seq data from GSE146115 datasets were analyzed. HERC2 gene counts >0 was identified as HERC2-positive cells. (A) UMAP plot of tumor cells grouped by HERC2 expression levels. (B) Feature plot of ALB-identified HCC cells. (C) Dot plot of gene expression levels from HALLMARK_IL6_JAK_STAT3_SIGNALING in GSEA database. (D) Dot plot of gene expression levels from chip-seq data in the GSEA database.

**Supplementary Figure 4**

**
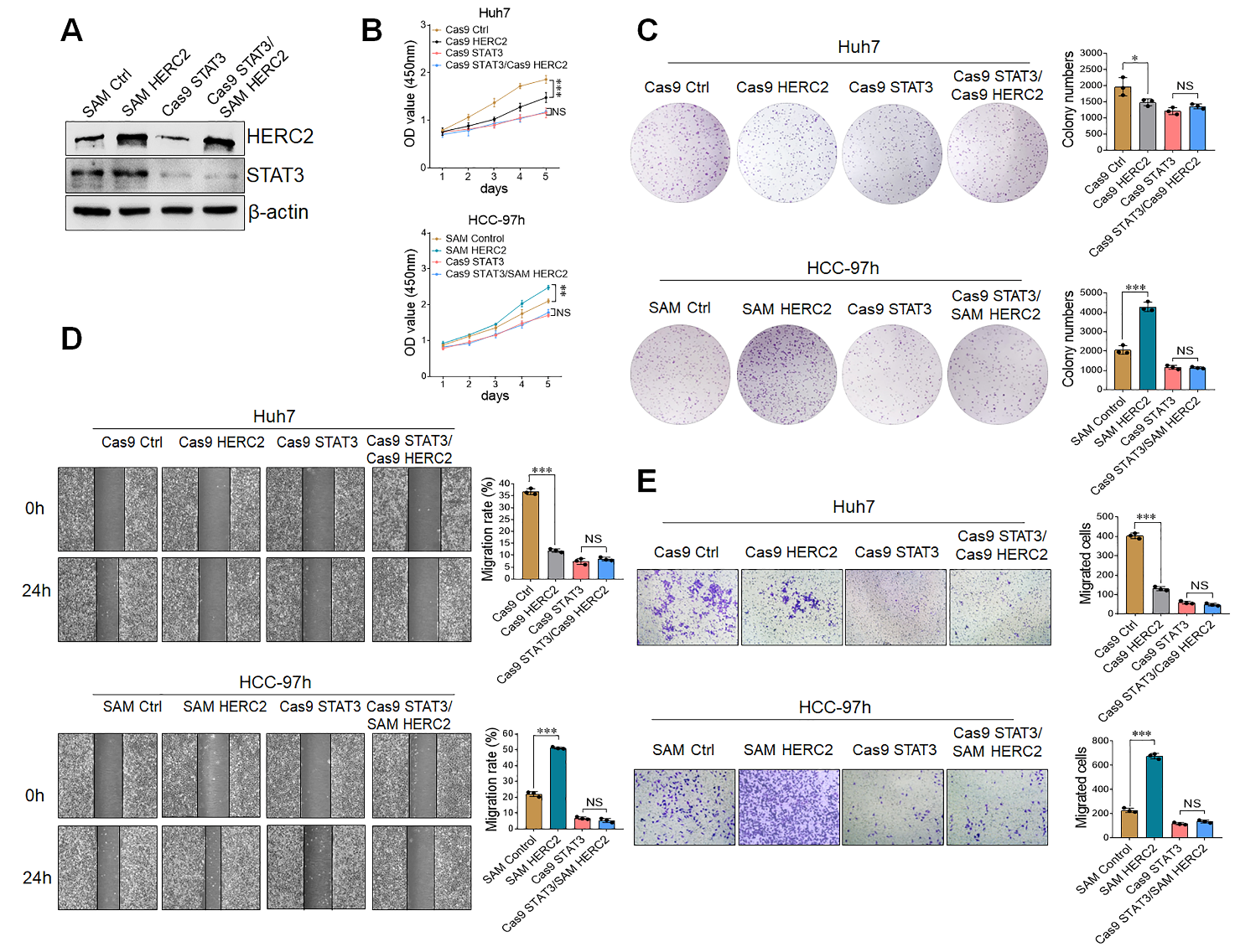
**

**Supplementary Fig. S4** **HERC2 promoted malignancy of HCC cells via STAT3 signaling**. (A) HERC2-overexpressing but STAT3-deficient HCC-97h cell lines were established. (B) A CCK-8 assay was used to detect cell proliferation. (C) A colony formation assay was used to evaluate cell proliferation. (D) A wound healing assay was used to detect the migration ability of the cells. (E) A migration test was performed to detect the migration ability of the cells. NS: not significant, *p<0.05, **p<0.01, ***p<0.001. Data from one representative experiment of three independent experiments are presented.

**Supplementary Figure 5**

**
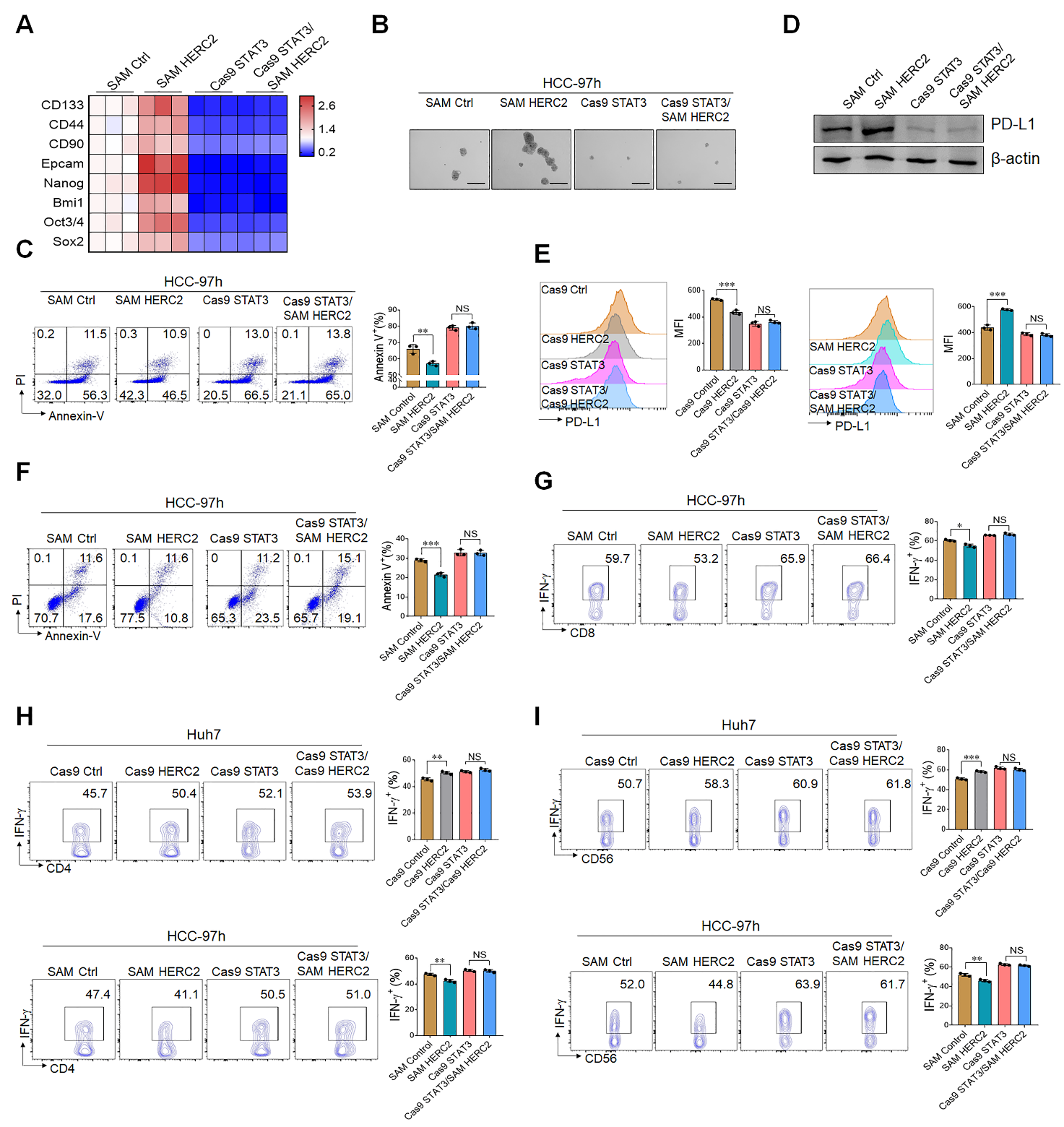
**

**Supplementary Fig. S5** **HERC2 enhanced stemness and immune evasion of HCC cells through STAT3 signaling**. (A) HERC2-overexpressing cells were treated with 50 ng/ml IL-6 for 24 hours. The RT-qPCR assay was used to detect the mRNA expression of cancer stem cell-related genes. (B) The cells were cultured in a conditioned medium with 100×N2, 50×B27, 20 ng/ml EGF, 10 nmol FGF, 5 μg/ml insulin, and 0.4% BSA for 7 days, scale bars=100μm. (C) The cells were treated with 20 μM sorafenib for 24 hours. A flow cytometry assay was used to determine the percentage of apoptotic cells. (D, E) The cells were treated with 50 ng/ml IL-6 for 24 hours. (D) The expression of PD-L1 was detected by western blotting assay. (E) Flow cytometry analysis was used to determine PD-L1 expression on cell surface. (F-I) Activated PBMCs were cocultured with HCC cells at the ratio of 4:1 for 24 hours. Apoptosis of HCC cells was detected by flow cytometry assay (F). IFN-γ levels of CD8^+^ T cells (G), CD4^+^ T cells (H) and CD56^+^ NK cells (I) were determined by flow cytometry analysis. NS: not significant, *p<0.05, **p<0.05, ***p<0.001. Data from one representative experiment of three independent experiments are presented.

**Supplementary Figure 6**

**
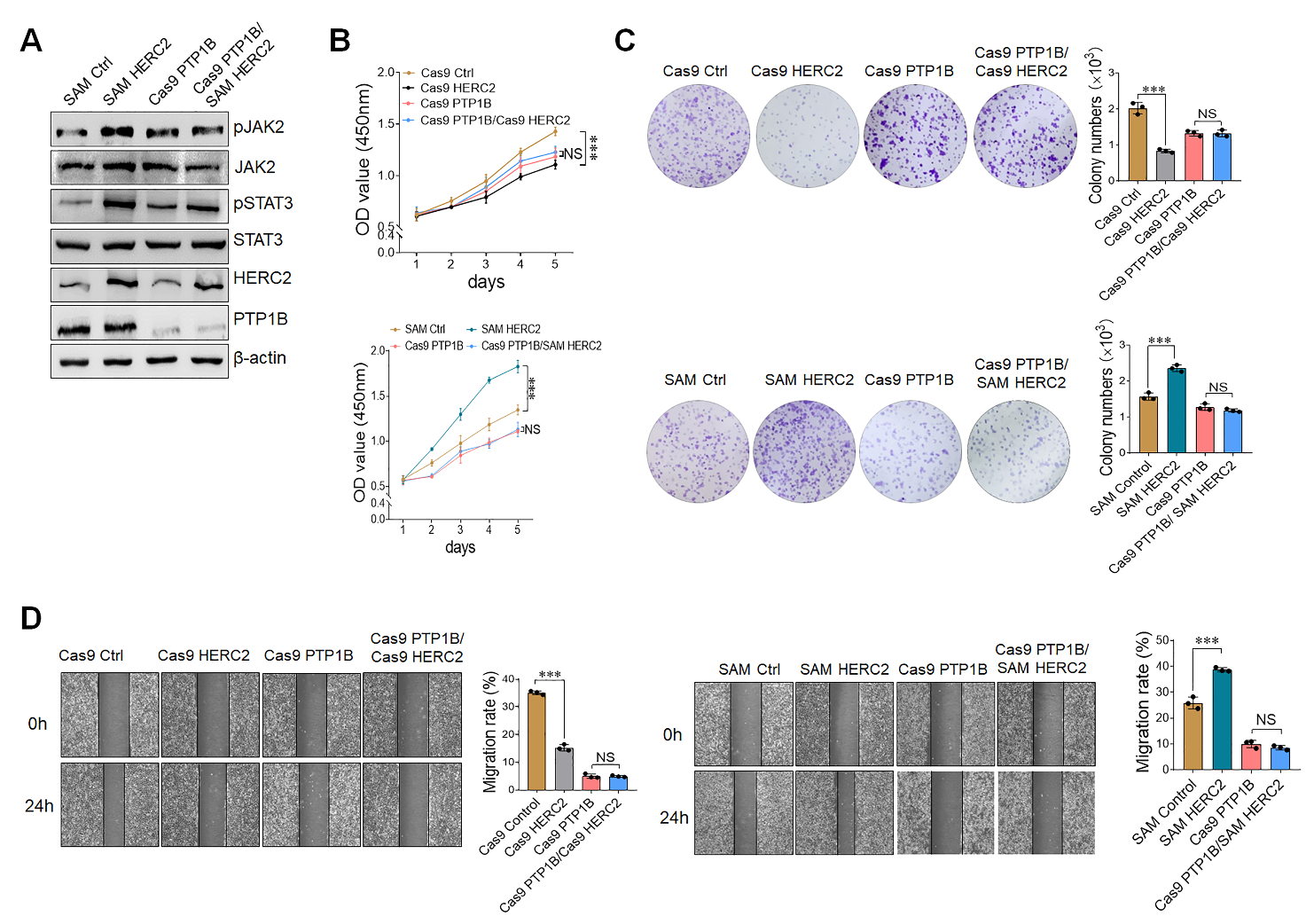
**

**Supplementary Fig. S6** **HERC2 promoted malignancy of HCC cells through PTP1B**. (A) HERC2-overexpressing and PTP1B-deficient HCC-97h cell lines were established. Cells were treated by 50ng/ml IL-6 for 20 min and JAK2-STAT3 signal activation was detected by western blotting assay. (B) A CCK-8 assay was used to detect cell proliferation in the cell lines. (C) A colony formation assay was used to investigate cell proliferation in the cell lines. (D) A wound healing assay was used to detect the migration ability of the cells. NS: not significant, ***p<0.001. Data from one representative experiment of three independent experiments are presented.

**Supplementary Figure 7**

**
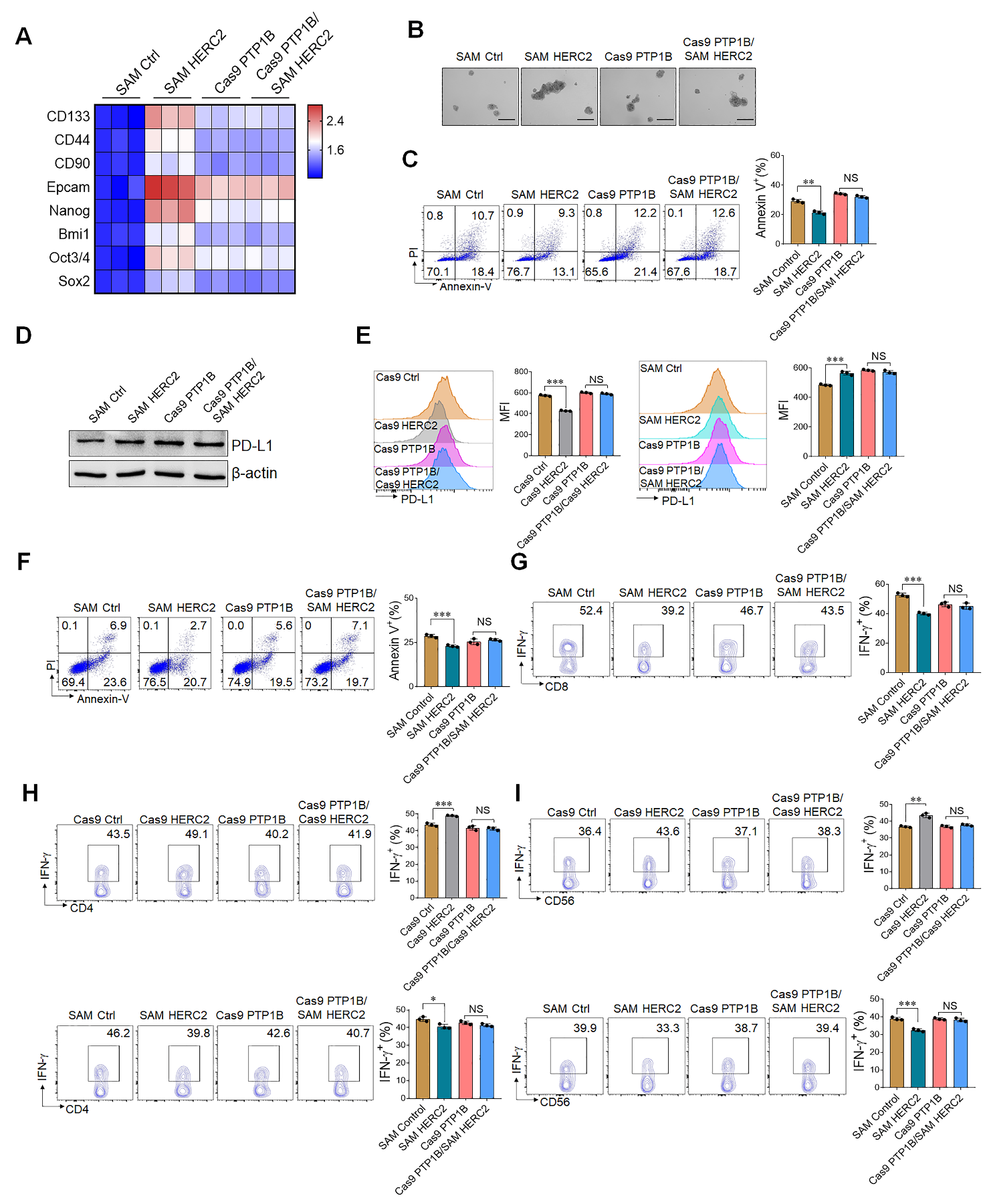
**

**Supplementary Fig. S7** **HERC2 promoted stemness and immune evasion of HCC cells through PTP1B**. (A) Cells were treated with 50 ng/ml IL-6 for 24 hours, and RT-qPCR assay was used to detect the mRNA expression of stemness-related genes. (B) Cells were cultured under 100×N2, 50×B27, 20 ng/ml EGF, 10 nmol FGF, 5 μg/ml insulin, and 0.4% BSA conditions for 7 days, scale bars=100μm. (C) Cells were treated with 20 μM sorafenib for 24 hours, and flow cytometry assay was performed to detect cell apoptosis. (D, E) The cells were treated with 50 ng/ml IL-6 for 24 hours. (D) The expression of PD-L1 was detected by western blotting assay. (E) Flow cytometry analysis was used to determine PD-L1 expression on the cell surface. (F-I) Activated PBMCs were cocultured with HCC cells at the ratio of 4:1 for 24 hours. Apoptosis of HCC cells was detected by flow cytometry assay (F). IFN-γ levels of CD8^+^ T cells (G), CD4^+^ T cells (H) and CD56^+^ NK cells (I) were determined by flow cytometry analysis. NS: not significant, *p<0.05, **p<0.01, ***p<0.001. Data from one representative experiment of three independent experiments are presented.

**Supplementary Figure 8**

**
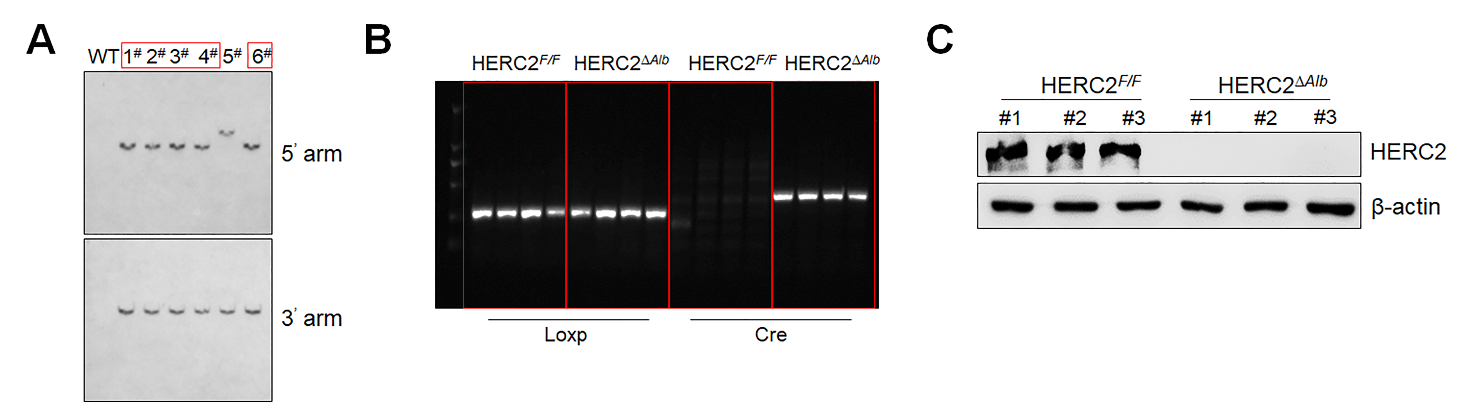
**

**Supplementary Fig. S8** **Establishment of hepatocyte-specific HERC2 knockout mice.** (A) Mouse embryonic stem cells were screened by Southern blotting analysis. (B) The mouse genotype was identified by PCR analysis. (C) HERC2 expression in primary mouse hepatocytes was evaluated by western blotting analysis.
